# The Diversity of Liquid Biopsies and Their Potential in Breast Cancer Management

**DOI:** 10.3390/cancers15225463

**Published:** 2023-11-17

**Authors:** Corinna Keup, Rainer Kimmig, Sabine Kasimir-Bauer

**Affiliations:** Department of Gynecology and Obstetrics, University Hospital of Essen, 45147 Essen, Germany

**Keywords:** liquid biopsy, blood, breast neoplasm, precision medicine, early detection of cancer, residual neoplasm, prognosis, genetic predictive testing, drug response biomarkers

## Abstract

**Simple Summary:**

In breast cancer patients, a blood sample contains components from tumor origin as well as those influenced by the tumor disease. Blood samples are being discussed as an early detection method and, under therapy, blood analysis was shown to have the potential of adapting therapy or to detect remaining breast cancer cells to forecast a recurrence. It is clear that blood components can forecast patients’ outcomes; however, blood samples for risk estimation are not used in clinical routine. In a subgroup of breast cancer patients, the detection of mutations in a specific gene using cell-free DNA from blood might be suitable for therapy monitoring. In this context, analysis of ESR1 and PIK3CA mutation detection in cfDNA has already been recommended to select targeted therapies. However, the usage of blood for therapy management still has challenges, like a lack of preanalytical and analytic standards and difficulties in proving the clinical utility.

**Abstract:**

Analyzing blood as a so-called liquid biopsy in breast cancer (BC) patients has the potential to adapt therapy management. Circulating tumor cells (CTCs), extracellular vesicles (EVs), cell-free DNA (cfDNA) and other blood components mirror the tumoral heterogeneity and could support a range of clinical decisions. Multi-cancer early detection tests utilizing blood are advancing but are not part of any clinical routine yet. Liquid biopsy analysis in the course of neoadjuvant therapy has potential for therapy (de)escalation.Minimal residual disease detection via serial cfDNA analysis is currently on its way. The prognostic value of blood analytes in early and metastatic BC is undisputable, but the value of these prognostic biomarkers for clinical management is controversial. An interventional trial confirmed a significant outcome benefit when therapy was changed in case of newly emerging cfDNA mutations under treatment and thus showed the clinical utility of cfDNA analysis for therapy monitoring. The analysis of PIK3CA or ESR1 variants in plasma of metastatic BC patients to prescribe targeted therapy with alpesilib or elacestrant has already arrived in clinical practice with FDA-approved tests available and is recommended by ASCO. The translation of more liquid biopsy applications into clinical practice is still pending due to a lack of knowledge of the analytes’ biology, lack of standards and difficulties in proving clinical utility.

## 1. Introduction

### 1.1. Breast Cancer

Over the last 30 years, the absolute breast cancer (BC) incidence rose in most countries [1] and worldwide totaled 2.3 million cases in 2020. In contrast to the rising incidence, the cumulative risk in mortality dropped from 2.5% to 1.65% (Germany)/1.5% (US) over the last 30 years [2]. The facts that life expectancy is increasing worldwide and the accumulation of somatic mutations increases with age partly explains the increasing cancer incidence. In 2005, it was stated that 10–15% of BC patients develop distant metastases within three years after the initial BC diagnosis [3].

Due to various molecular aberrations, BC presents as a highly heterogeneous disease and sub-typing the molecular characteristics of each BC case is important [4]. Molecular characterization of the disease by estrogen receptor (ER), progesteron receptor (PR) and HER2 protein staining on the tumor tissue is the gold standard [5,6]. This approach leads to distinguishing (a) hormone receptor (HR)-positive, HER2-negative BC patients, (b) HR-positive, HER2-positive patients, (c) HR-negative, HER2-positive patients and (d) HR-negative and HER2-negative (triple negative BC, TNBC) patients [7]. As a more comprehensive analysis of the heterogeneous tumor biology, the PAM50 assay was proposed, which analyzes the gene expression of 50 genes in tumor tissue and thus divides breast cancer patients into luminal A, luminal B, HER2-enriched, and basal-like patients by their "intrinsic" subtypes [8]. The stratification of the BC subtypes becomes particularly plausible when considering the differences in the overall survival (OS) data of the different BC subtypes as well as the different treatment options for the BC subtypes. The German BC registry (TMK) cohort study included 1395 BC patients from February 2007 until October 2015 with HER2-positive patients showing a mean OS of 38.2 months, hormone receptor (HR) positive and HER2-negative patients of 33.8 months and triple-negative BC (TNBC) patients of only 16.8 months [9]. The targeted therapy options are for HER2-positive and also HR-positive patients, but a lack of tailored therapy options for TNBC patients might account for these survival data.

HR-positive BC patients receive anti-hormonal therapy, known as endocrine therapy. HER2-positive BC patients are treated with anti-HER2 treatment. Further targeted therapy options include PARP inhibition for patients with germline BRCA1/2 mutations and PI3K inhibitors for patients with somatic PIK3CA mutations. HR-positive/HER2-negative metastatic BC patients are currently treated with CDK4/6 inhibition as the first-line therapy. In TNBC patients with specific biomarker status, immune checkpoint inhibition can be prescribed. In this review, only liquid biopsy studies referring to BC are discussed—in Section 8, the usage of blood components for the selection of targeted therapies in BC is discussed.

### 1.2. Liquid Biopsy (LB)

Molecular characterization of BC by tissue biopsy is fraught with several limitations. Tissue biopsies do not represent the spatial heterogeneity. In addition, tumor evolution resulting in temporal heterogeneity needs consideration for treatment decisions, thus, molecular real-time snapshots of the disease would be optimal for therapy guidance. However, sequential tissue biopsies are not convenient and often not feasible. The analysis of body fluids, such as blood, mirrors the spatial heterogeneity and by repeatable sampling, due to a minimal-invasive blood draw, the temporal heterogeneity is also mirrored. Consequently, liquid biopsy (LB) enables tracking the individual BC plasticity with the potential for patient-based targeted BC management.

**Figure 1:**
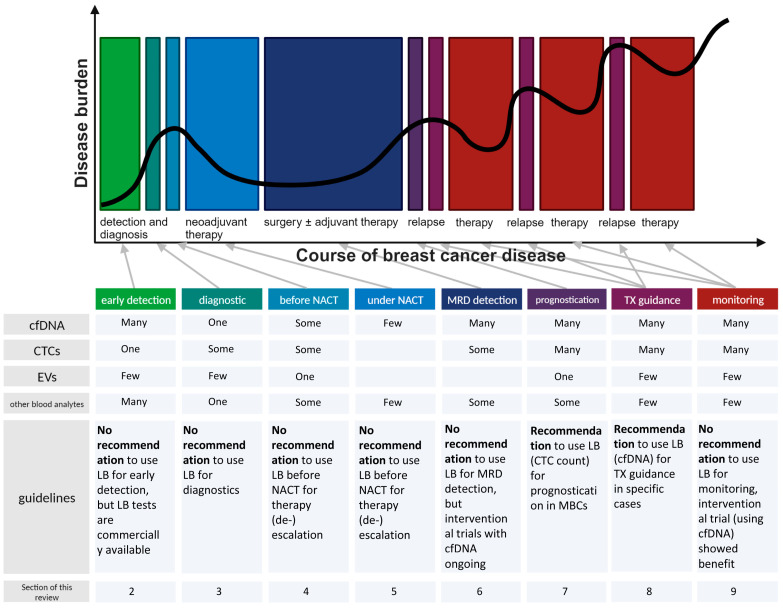
Liquid Biopsies for therapy management of BC patients. Different liquid biopsy analytes were evaluated for their suitability for clinical applications over the entire course of the BC disease. The number of references studying the specific blood analytes for the specific clinical BC application is approximated. The sections of this review article discussing the different applications are listed. As the conclusion of this review article, the guidelines and recommendations for the usage of liquid biopsy in each application are summarized. Created with BioRender.com. Abbreviations: cfDNA: cell-free DNA; CTCs: circulating tumor cell: EVs: extracellular vesicle; LB: liquid biopsy; MBC: metastatic breast cancer; MRD: minimal residual disease; NACT: neoadjuvant chemotherapy; TX: therapy.

Conquering the limitations associated with tumor heterogeneity and acquired resistance, LB is useful in all stages of the disease (Figure 1). The early detection of BC by population screening using blood analysis is one field of application as well as the detailed diagnosis and sub-typing of the disease (Section 2 and Section 3). After BC detection and diagnosis, prognostication via LB might be used to (de)escalate the intervention (Section 4 and Section 7). Monitoring of response during treatment via LB might as well be suitable for therapy (de-)escalation or treatment adaptations (Section 5 and Section 9). Especially in the BC setting, the identification of minimal residual disease is a major field for the application of serial liquid biopsies (Section 6). Finally, the minimal-invasive acquisition of real-time information by blood analysis has the power to tailor and individualize therapies precisely for targeted intervention to prolong survival along with assurance of optimal quality of life (Section 8).

In BC, LB analysis is mostly conducted using blood. Within this complex composed body fluid, a diversity of analytes exists, referred to as circulome. In the past and mostly also at present, single blood analytes were and are analyzed. However, the realization of the variety of blood analytes recently led to the establishment of multimodal analyses with more than a single blood component from the same blood sample, almost all of which showed an additive value of multiparametric profiling [10] as reviewed last year [11,12].

In the oncological setting, circulating tumor cells (CTCs), tumor-derived extracellular vesicles (EVs), cell-free DNA (cfDNA) or RNA (cfRNA) and in particular circulating tumor DNA (ctDNA) or RNA (ctRNA) and proteins should be mentioned in this incomplete list of potential analytes.

#### 1.2.1. cfDNA

The majority of cell-free DNA is derived from hematopoietic, non-neoplastic cells undergoing apoptotic cell death [13,14,15] and has already been described in healthy donors in 1948 [16]. Necrosis, other mechanisms of cell death and active secretion are also discussed as cfDNA release mechanisms [17]. Due to the fact that cfDNA is largely derived from dying cells, cfDNA is an indicator of cellular turnover [18]. Increased cellular turnover is a characteristic of neoplasms, thus, in tumor patients a proportion of cfDNA derives from the tumor. In 1977, it was shown that the amount of cfDNA in the blood of tumor patients was higher than in healthy subjects and the difference was even greater in metastatic tumor patients compared to healthy subjects [19]. Although evidence increased over time that the mutant allele fraction and the amount of cfDNA directly correlated with tumor volume [20], it became obvious that cfDNA concentration was highly dependent on the biological context and lifestyle factors, such as stress, chronic inflammation, physical activity, nutrition and smoking [21]. In addition to the great variability and relatively low abundance, cfDNA is rapidly cleared [22,23] and highly fragmented with size distributions corresponding to nucleosome-associated DNA [24].

#### 1.2.2. CTC

Especially in BC, tumor cells can invade the bloodstream in very early stages [25]. Invasion into and survival within the circulation is mediated by significant changes in the phenotype called epithelial-to-mesenchymal transition (EMT) [26] and also by immune evasion mechanisms [27]. Some CTCs have stem cell properties [28,29,30] and are regarded as metastatic precursors [31]. As CTCs are part of the metastatic cascade, their characteristics are of relevance for oncological management. The advantage of CTCs compared to other blood analytes is the possibility to examine their cellular character with genomic, transcriptomic, proteomic, metabolic and phenotypic features. Single-cell resolution depicts the heterogeneity and subclonal evolution [32,33], but a large number of single CTCs is to be analyzed to identify the range of heterogeneity within a systemic oncologic disease [34]. While some studies state that cfDNA mutations accurately reflect the mutations detected in a single CTC [35], others conclude emerging resistance mutations to be earlier detectable in CTCs than cfDNA [34].

#### 1.2.3. EV

EVs are lipid-bilayer-enclosed compartments released by malignant and non-malignant cells [36]. EVs in the circulation mainly originate from hematopoietic, non-neoplastic cells [37]. Tumor-derived EVs can be detected in the blood of cancer patients and thus, EV analysis in the plasma can provide insights into the systemic oncologic setting, including the changes in the immune system [38]. EVs contain molecules from the cell of origin, mostly RNAs and proteins [39]. In line with the heterogeneity of release mechanisms, cells of origin and cargo, EVs, enabling intercellular communication [40], were shown to have implications in various cellular processes [41], like pre-metastatic niche formation [42]. Transferred mRNA and microRNA (miRNA) have been reported to change the gene expression in the recipient cell [43], while the protein content within the EVs or on their surface modulates the signaling pathways of recipient or interacting cells [44].

#### 1.2.4. Analytical Dimensions

Considering the various blood components insightful for oncological management, it is important to note that the analytical dimensions range broadly depending on the clinical question and blood analyte. The quantification of different blood analytes such as CTC number, cfDNA concentration, mean ctDNA fraction, cfRNA copies and protein abundance is informative. DNA analyses can include mutation detection, but also detection of other genomic alterations, like fusions, insertions, deletions and copy number alterations. DNA methylation is an additional dimension most relevant for tissue-specific results and with implications for gene expression of the cell of origin. Fragment size, end motives and topology can also be analyzed in cfDNA [45]. Besides quantification of the most different RNA species and gene expression measurement, the identification of splice variants is an additional dimension on the transcriptional level. Protein activity, isoforms, localization, topology, and post-translational modifications are additional dimensions for LB analysis. Phenotypic characterization reveals another dimension for LB analysis.

## 2. Liquid Biopsies for Early Breast Cancer Detection

Mammography is the established gold standard for BC screening in clinical practice; however, it is restricted to a certain age group of persons. Currently, BC is mostly diagnosed when symptoms are observed. Recently, blood-based tests for multi-cancer early detection (MCED) were developed for persons of all ages and are usable to screen the general population for multiple cancer entities.

### 2.1. cfDNA

The first blood-based MCED test results were published in 2018 (Table 1) showing a specificity of 99% and a sensitivity of 33% to detect eight tumor types, including BC, by targeted cfDNA mutation analysis combined with the evaluation of circulating proteins—the CancerSEEK test [46].

In 2016, GRAIL started the Circulating Cell-free Genome Atlas Study (CCGA, NCT02889978). In the sub-study 1, whole genome bisulphite sequencing for methylation analysis and targeted sequencing single nucleotide variants with paired white blood cell background removal were selected as the best cfDNA analysis methods [47] and thus, were used for the CCGA sub-study 2 and the STRIVE study (NCT03085888) [48]. The specificity was 99.3% and the sensitivity to detect BCs with stage II disease was around 50% (Table 1). Among all cancers and stages, the tissue of origin accuracy was reported to be >75%, in the validation cohort for BC even 93% [48]. In the CCGA sub-study 3, using targeted methylation cfDNA sequencing panel [49], specificity was 99.5% and sensitivity for BC was only 30.5% across all stages. Application of this CCGA sub-study 3 in patients with symptoms only showed an increased sensitivity of 52.8% for BC [50].

In 2019, GRAIL started the PATHFINDER study (NCT04241796) to evaluate the integration of a targeted cfDNA methylation-based MCED test (MCED-Scr; 30,000 CpG fragments covered [51]), here referred to as Galleri, [52] by implementation in adults with elevated cancer risk. At ESMO (European Society for Medical Oncology) Congress 2022, it was reported that adding Galleri test to the standard of care screening more than doubled the number of cancers detected. As over 50 cancer entities can be detected, 71% of participants with Galleri-detected cancer had cancer types with no routine screening test available. The positive predictive value (PPV) was 43.1% and the false-positive rate was less than 1%. Since July 2022, the Galleri MCED-Scr by GRAIL is commercially available however, not covered by insurance.

In the discussion about blood-based MCED tests, many aspects are in consideration. The specificity has to be high to minimize false-positive results as they would have an unnecessary psychological impact and unnecessary radiation dose due to the follow-up scans. A plan should exist for how to proceed with subjects with a positive test result and with the subjects with non-identifiable tumors in the follow-up scans, as in the NCI study Vanguard. In this context, it is important to note that specificity is hampered by alterations accumulating in the hematopoietic system during aging. For population screening, sensitivity must be high because of the low incidence of cancers in the general population. The most important parameter is the PPV, representing the chance of somebody with cancer having a positive test. The PPV is dependent on the parameters sensitivity, specificity and disease prevalence within the screened population. Especially in the studies with subjects with symptoms, the PPV is overestimated and would be <10% for the current Galleri test in the general population [51]. Third, until now, clinical utility has not been shown for MCED testing. It is still unknown whether these tests will shift time of detection of the disease from the late stages to earlier stages and whether achieved early-stage diagnoses might be early enough to allow curative treatment, reduce mortality and prolong survival [53]. At the moment, data only depict early stage cancers to be detectable with low sensitivity [48]. Low sensitivity and low PPV, as well as uncertainty about the clinical utility in terms of reduced mortality currently prohibit the usage of a blood-based MCED test for population screening.

### 2.2. cfDNA and Other Analytes Proposed by Small Non-Interventional Trials

Despite the current sobering state of blood-based MCED testing in clinical practice and the data of huge clinical trials, some small non-interventional studies have shown certain blood analytes and dimensions to be potentially useful for BC detection (Table 1). Regarding single analyte and single dimension studies for BC detection, cfDNA concentration [54], cfDNA *PIK3CA* mutations [55], cfDNA methylation [56,57,58,59,60,61], and cfDNA integrity index [62] should be mentioned. Even the detection of cfDNA mutations in breast milk to detect BC postpartum was recently suggested [63]. Detection of CTCs [64], EVs with specific proteomic profiles [65,66], EVs including unique tRNAs and/or miRNAs [67,68], circulating miRNAs [69,70,71], a long non-coding RNA [72], proteins [73,74,75] and volatile organic compounds in the urine [76] were also discussed as potential BC detection markers.

Referring to the multimodal approach of the CancerSEEK test, it was recently discussed whether a multimodal blood-based MCED approach with cfDNA mutation and cfDNA methylation as well as circulating miRNA information would increase the sensitivity of cancer detection [77]. Even though the Gallergi test is only based on targeted cfDNA methylation, it was stated in the CCGA sub-study 1 that pan-cancer features, combining more than one dimension, yielded the lowest limit of detection [47]. Low-coverage whole genome sequencing (WGS) for cfDNA fragmentation pattern analysis combined with high-coverage targeted cfDNA sequencing for mutation analysis was also suggested for BC early detection [78]. The cfDNA integrity might, in combination with the detection of CTCs, also be suitable for BC detection [79]. A combination of circulating mRNAs and a protein [80] might also be useful for BC detection.

To conclude, BC detection by analysis and combination of various LB components is promising. However, a blood-based BC detection test is not part of any clinical routine, as the PPV has to be increased and the clinical utility proven first. Until then, only self-examination and mammography are used for population screening and confirmation of the diagnosis is only possible by means of cyto- and histopathological criteria [81].

## 3. Liquid Biopsies for Detailed BC Diagnostic

After BC detection, a comprehensive characterization of the disease is required for tailored BC management. Some LB approaches for BC subtype characterization, staging of the disease and identification of the histology are under evaluation. Subtyping is currently conducted via protein analysis on the tumor tissue regarding ER, PR and HER2 protein expression. This evaluation leads to the differentiation of (a) HR-positive, HER2-negative BC patients, (b) HR-positive, HER2-positive patients, (c) HR-negative, HER2-positive patients and (d) HR-negative and HER2-negative (triple negative BC, TNBC) patients [7]. It has already been shown that ‘intrinsic’ subtypes can be determined by the PAM50 test, which evaluates the gene expression of 50 genes in the tumor tissue [8]. A recent study examined the profiling of the PAM50 transcripts not in the tissue, but in EVs and concluded a good concordance to the tissue results [82]. As it is possible to indirectly determine the gene expression by cfDNA sequencing read death [83], it is to question whether the PAM50 test might, in principle, be transferred to cfDNA material as well. It was recently shown that the PAM50 signatures from tissue RNA expression data correlated to the BC subtype signatures evaluated by matched ctDNA copy number analysis [84].

### 3.1. cfDNA and Nucleosomes

Recently, the analysis of the nucleosome position and accessibility of cfDNA by WGS with 0.1x coverage was reported to differentiate ER-positive from ER-negative MBCs [85] (Table 2). BC subgroups were also shown to be differentiable on the basis of CNV analysis on the tumor tissue [86]. This approach is transferable to cfDNA analysis [84], especially in samples with a high ctDNA fraction [87]. High ctDNA fraction itself has already been shown to correlate with TNBC status, and also high tumor grade and metastatic status [88].

### 3.2. Multimodal LB

One multimodal LB approach to subtype MBC is the DefineMBC approach by Epic Science (San Diego, CA, USA). In addition to the 56-gene cfDNA Panel (including SNV, CNV, MSI, TMB analysis) that has been covered by the MediCare program in the US since April 2023 [89], CTCs are also analyzed regarding ER and HER2 protein expression and ERBB2 amplification.

### 3.3. Circulating RNA in or Independent of EVs

Interestingly, also the miRNA cargo within EVs can differentiate the BC subtypes. EV miRNA-373 was increased in the blood of TNBCs patients compared to patients with other BC subtypes [90]. While a panel of 45 miRNAs detected in plasma EVs of BC patients differentiated HER2-positive from TNBC patients [91]. Despite BC subtyping, EV miRNAs might also be used to determine BC stage, as MBCs were shown to have significantly higher EV miR-21 levels compared to BC patients with no metastases [92]. EV miR-223-3p was significantly increased in invasive ductal carcinoma patients compared to subjects with ductal carcinoma in situ (DCIS) [93]. Circular RNA, circulating in the blood, specifically circ_0001785, is proposed in a small cohort study to be correlated with distant metastasis and histology [94].

### 3.4. CTCs

Analyzing CTCs might as well be used for detailed BC diagnosis. One widely used CTC isolation platform is the CellSearch (Menarini Silicon Biosystems, Huntington Valley, PA, USA) system, which captures cells based on the surface expression of the epithelial marker EpCAM and cells are identified as CTCs when stained positive for Cytokeratin (CK) and negative for CD45. A significantly increased number of CTCs, determined by CellSearch, before therapy was reported for MBCs with lobular compared to ductal histology [95]. Furthermore, androgen receptor (AR) expression on CTCs was correlated with bone metastasis [96] and a higher number of apoptotic CTCs was detected early in contrast to metastatic BC patients [97].

## 4. Liquid Biopsies before (Neo)Adjuvant Treatment for Therapy (De)Escalation

In the following and in Table 3, the prognostic value of LB analysis in BC patients before starting (neo)adjuvant treatment to forecast the achievement of a pathological complete remission (pCR), risk of recurrence or disease-free, BC specific and overall survival are discussed. Prognostication can guide therapy decisions as to (de)escalate the treatment regimens.

### 4.1. Tissue Analysis

With an Oxford level of evidence (LOE) of 2a, the analysis of tumor infiltrating lymphocytes (TILs) in the tumor tissue is recommended by the German working group gynecological oncology for BC patients before neoadjuvant treatment as prognostic marker for achieving a pCR [98]. Translating this knowledge into the field of LB, it has been shown in a meta-analysis including more than 17,000 patients with early BC, that high pre-treatment neutrophil-to-lymphocyte ratio (NLR) is linked to poor prognosis [99] and thus, it was concluded that a more aggressive treatment to prevent a recurrence is needed in early BC patients with high pre-treatment NLR. Other studies validated this result and drew the same conclusions [100,101,102,103].

With an Oxford LOE 2b, KI67 protein expression analysis on the tumor tissue before neoadjuvant treatment also has prognostic value while the presence of germline BRCA mutations before chemotherapy initiation forecasts higher probability to achieve a pCR and both analyzes are recommended to (de)escalate therapy regimens [98]. The latter evaluation can be conducted by blood-based analysis.

In clinical practice, gene expression profiling of the tumor tissue before neoadjuvant treatment (commercially available as f.e. Mammaprint^®^, Endopredict^®^, OncoType Dx^®^, Prosigna^®^ and Breast Cancer Index^SM^) has prognostic value and is used to select for optimal therapy regimens [98]. Until now, indirect gene expression profiling from blood before neoadjuvant treatment has not yet been used in clinical practice.

Multiple LB analytes with a potential for prognostication before treatment initiation in early BC exist.

### 4.2. cfDNA

A meta-analysis has proven the prognostic role of ctDNA quantity pre-neoadjuvant treatment to predict the risk of relapse and OS [104]. One study analyzed the value of cfDNA quantity before neoadjuvant chemotherapy (NACT) in TNBC patients and concluded that cfDNA was independently predicting the risk for recurrence [105]. Within the translational analysis of the NeoALTTO trial, *PIK3CA* and/or *TP53* mutation detection in cfDNA before neoadjuvant anti-HER2 treatment correlated with lower pCR rates in HER2-positive BC patients [106], thus, HER2-positive patients with undetectable *PIK3CA* and *TP53* mutations are proposed as best candidates for treatment deescalation strategies. In addition to the cfDNA mutation analysis, the evaluation of cfDNA methylation of GASTP1, RASSF1A and RARB2 before neoadjuvant treatment in early BC patients was associated with OS independent of pCR [107].

However, in 2023, the German working group gynecological oncology did not recommend the usage of cfDNA isolated from peripheral blood as a prognostic marker before neoadjuvant therapy, although Oxford LOE was 2a [98].

### 4.3. CTCs

CTC detection by CellSearch at baseline of the NeoALTTO trial in HER2-positive BC patients receiving neoadjuvant anti-HER2 therapy resulted in numerically lower pCR rates [108]. In the GeparQuattro trial, CTC detection and achievement of a pCR was not evaluated, but CTC detection before NACT correlated significantly with disease-free (DSF) and OS, although in different sub-cohorts different CTC cut-offs were applied [109]. In the BEVERLY-1 and -2 trials, CTC quantity by CellSearch analysis before start of NACT did not show any correlation to pCR rates, but CTC detection was associated with significantly decreased DFS and OS [110].

Two groups performed a meta-analysis of studies including in total more than 2000 early BC patients [111,112]. Both groups concluded that the presence of CTCs before neoadjuvant treatment was an independent predictor of poor DSF, distant disease free (DDSF) and OS. Despite this evidence evaluated as Oxford LOE 1b, the German working group gynecological oncology did not recommend the usage of CTCs as prognostic marker before neoadjuvant therapy in 2023 [98]. Although not evaluated before neoadjuvant therapy, but before adjuvant therapy in the SUCCESS trial, it is important to note that CTC detection in this setting was also significantly associated with poor DSF and OS [113].

### 4.4. EVs and miRNAs

The characterization of EVs isolated from plasma drawn before neoadjuvant therapy in BC patients revealed levels of specific EV miRNA to forecast pCR [114]. Even miRNAs directly found in the plasma (not stated whether associated with EVs or not), were shown to correlate with relapse and OS in TNBC patients before neoadjuvant therapy [115] or with pCR in the entirety of BC subgroups receiving neoadjuvant treatment [116,117]. More specifically, only reduced miR-145 levels were related to pCR in HER2-positive BC, while let7a correlated with pCR in luminal BC patients [118]. Finally, even the level of circulating nucleosomes before neoadjuvant therapy had prognostic value in early BC patients [119].

## 5. Liquid Biopsies under Neoadjuvant Therapy for Therapy Switch/(De)Escalation

Blood-based analysis during therapy can be used as a monitoring approach to guide therapy switch and/or therapy (de)escalation after some cycles of neoadjuvant treatment. Although LB-informed neoadjuvant therapy adaptation has not been shown in large clinical trials to improve patient outcomes, some findings seem to hint in this direction (Table 3).

While the sole analysis of miR-141, miR-34a, miR-182 and miR-183 in EVs after the first dose of neoadjuvant therapy predicted pCR/non-pCR [114], increased levels of circulating miR-148a-3p and miR-374a-5p from baseline to two weeks of trastuzumab-based neoadjuvant therapy in the NeoALTTO trial were related to pCR [120]. Evaluation of thymidine kinase activity in the plasma early under neoadjuvant treatment was also shown to have prognostic value [121]. Within the I-SPY 2 study evaluating the effect of neoadjuvant therapy with an AKT inhibitor in 84 high risk early BC patients, the ctDNA clearance from baseline to three weeks after therapy initiation was related to an increased pCR rate (48% pCR compared to 17% pCR in patients with ctDNA after three weeks) and ctDNA presence after 3 weeks under therapy was significantly associated with increased risk of metastatic recurrence (HR 4.5; 95% CI 1.2–17.4) [122]. Detection of mutations in cfDNA based on tumor-informed personalized assays under neoadjuvant therapy were correlated with a lower chance of pCR [123].

## 6. Liquid Biopsies to Anticipate Minimal Residual Disease

In early BCs treated with neoadjuvant therapy, pCR detection is prognostic and currently influences the consecutive therapy approaches. However, not all patients not achieving a pCR have a poor prognosis and, vice versa, some patients with pCR relapse. Consequently, we need biomarkers (Table 4) to identify patients at risk to offer additional therapy or therapy escalation to finally improve patients’ outcomes.

Currently, no LB test is applied in clinical practice to detect patients at risk after neoadjuvant therapy, surgery, during adjuvant therapy or in the follow-up. However, several trials are ongoing aiming to integrate blood-based analysis for identification of patients with minimal residual disease (MRD). The goal for upcoming LB assays is to antedate even relapses that occur later than two years after neoadjuvant treatment/surgery, so that positive assay results could indicate therapy escalation, but negative assays could even indicate treatment de-escalation.

### 6.1. Persistence of LB Signals under Neoadjuvant Treatment to Anticipate MRD

#### 6.1.1. cfDNA

ctDNA clearance during neoadjuvant therapy was informative regarding the existence of MRD [124]. In this context, in the I-SPY 2 trial, cfDNA clearance from baseline to the end of treatment correlated with a pCR [122]. In general, patients with ctDNA detection after therapy showed a significantly increased risk for metastatic recurrence (HR 11.5; 95% CI 2.9–46.1). Patients not achieving a pCR but with no ctDNA detection after therapy had an especially excellent outcome, similar to the patients that achieved a pCR (HR 1.4; 95% CI 0.15–13.5) [122], indicating an additive value of LB analysis and pCR evaluation. In the I-SPY 2 trial, ctDNA detection was conducted by personalized mutation assays. Another study, utilizing a targeted digital sequencing approach to improve sensitivity in mutation detection, concluded that patients with a pCR showed a larger decrease in ctDNA during neoadjuvant therapy compared to the patients with no pCR [123]. In addition, ctDNA persistence even after neoadjuvant therapy detected by BC-specific methylation pattern indicated the existence of MRD [125]. In addition to the comparison of cfDNA mutation and methylation results at baseline and after neoadjuvant therapy, longitudinal cfDNA integrity analysis was also shown to be suitable to indicate tumor shrinkage and reduced Ki67 levels in case the cfDNA integrity increased [79]. Decreasing cfDNA integrity indices after neoadjuvant therapy correlated with the number of metastatic lymph nodes [79].

#### 6.1.2. CTCs

Regarding CTC analysis after neoadjuvant therapy, the presence of persisting CTCs correlated with shorter DSF and OS [113] and correlated with an increased risk of relapse [126]. 

Despite persistent cfDNA and CTC results under neoadjuvant treatment, circulating miRNA evaluation under neoadjuvant treatment might be used to anticipate MRD (Table 4). Persisting high levels of circulating miR-21 after neoadjuvant treatment were also associated with poor prognosis in two independent studies [92,117].

### 6.2. Liquid Biopsies after Neoadjuvant Treatment to Anticipate MRD

The sole analysis of blood after neoadjuvant treatment was shown, besides pCR, to identify patients with worse outcome and thus, was selected for intensified ‘post-neoadjuvant’ treatment (Table 4).

#### 6.2.1. cfDNA

Patients who achieved a pCR, but showed an reduced cfDNA integrity index after neoadjuvant therapy had a higher risk for distant metastases [79].

Evidence is compelling for the prognostic value of ctDNA detection by mutation analysis in all BC subgroups after neoadjuvant therapy [104,127,128,129,130,131]. However, the sensitivity of a single cfDNA mutation assessment after neoadjuvant therapy is low, so only a few patients with MRD can clearly be differentiated from patients who never will recur [130]. Serial sampling in the follow-up might be the solution for the low MRD detection sensitivity directly after neoadjuvant therapy, as discussed later in this section.

Additionally, ctDNA presence after neoadjuvant therapy was detected in 12/13 patients with no pCR, but also in 5/9 patients achieving a pCR [123]. The value of ctDNA detection after neoadjuvant therapy is still unclear since no follow-up data are available but might be in part additive to the information about pCR for MRD detection. On the other hand, in this study, ctDNA concentration but not ctDNA presence after neoadjuvant therapy was significantly correlated with a pCR [123]. Similar conclusions were drawn from the exploratory analysis of ctDNA samples in the phase III IMPASSION031 trial [132]. After neoadjuvant therapy using atezolizumab, TNBC patients achieving a pCR and who had no detectable ctDNA showed the best DSF and OS while the non-pCR cohort could be differentiated by ctDNA presence in patients with increased DSF and OS (ctDNA negative) and patients with worse DSF and OS (ctDNA positive) [132].

In early BC patients not receiving neoadjuvant therapy, ctDNA mutation analysis before surgery was shown to correlate with DSF and also with biologically aggressive phenotypes [133].

#### 6.2.2. CTCs

While one study showed that one or more CTCs present after NACT predicted relapse and survival in TNBC patients [126], another study using a different CTC enrichment method found no significant prognostic value for CTC analysis in TNBC patients after neoadjuvant therapy [131]. The molecular characterization of CTCs, for example, the evaluation of TWIST transcripts in CTCs [134], might be more precise in prognostication of DSF in early BC patients after surgery and before adjuvant therapy compared to CTC quantification.

In line with the additive value of multimodal LB analysis mentioned elsewhere, the usefulness of combining cfDNA and CTC analysis was also demonstrated for BC MRD detection [131]. The FoundationOne Liquid and FoundationACT liquid biopsy assays as well as a microfluidic platform were used to detect ctDNA and/or CTCs in blood drawn from TNBC patients after neoadjuvant treatment. MRD sensitivity was 79% with ctDNA analysis alone, 62% with CTC analysis alone and 90% with the combination of both analytes, respectively. The additive value of CTCs and ctDNA implicate the lack in correlation of these two analytes, thus, patients do exist that have a positive result in only one of the two analytes [131]. The high sensitivity for MRD detection utilizing CTCs and cfDNA might be used not only for therapy escalation but here even for therapy de-escalation in the future.

#### 6.2.3. Other Analytes

Despite cfDNA or CTC analysis, other blood analytes were evaluated for MRD detection. For example, the lymphocyte-to-monocyte ratio was shown to be significantly associated with worse prognosis in BC patients after surgery and neoadjuvant therapy [135]. Independent of neoadjuvant treatment but at the time point of surgery, the expression of TLR4 on peripheral blood mononuclear cells (PBMCs) predicted high risk of relapse in early BC patients [136]. Translational analysis of plasma samples after completion of neoadjuvant therapy in the NeoALTTO trial concluded a three-miRNA signature composed of miR-185-5p, miR-146a-5p and miR-22-3p to be a prognostic marker independent of pCR [137].

#### 6.2.4. Interventional Trials

Currently, three ongoing clinical trials include patients for post-neoadjuvant therapy, as a therapy escalation strategy, based on cfDNA results after surgery. In all three trials, only ctDNA-positive patients were included for therapy escalation, even though many patients had to be screened for this approach. In these and upcoming trials, the optimal time point for blood draw, potentially different subtype-specific ctDNA kinetics, tumor-agnostic versus tumor-informed strategies, the optimal mutation assay and optimal trial endpoints should be evaluated.

In ER-positive early BC patients with detectable ctDNA after surgery via the highly sensitive Signatera mutation assay, the investigator-initiated, multi-center, Phase II randomized LEADER trial (NCT03285412) evaluates the use of endocrine therapy with and without Ribociclib, a CDK4/6 inhibitor, in the adjuvant setting regarding the ctDNA clearance after one year and as secondary endpoint also regarding DFS.

The same ctDNA assay, Natera’s signatera test, is also used in the ZEST trial (NCT04915755) for HER2-negative and BRCA-mutated BCs or BRCA wildtype TNBCs. Only patients with a ctDNA positive result, independent of their pCR status, after surgery or adjuvant therapy, receive Niraparib, a PARP inhibitor and DSF is the primary endpoint. Currently, a trial recruiting early TNBCs for adjuvant capecitabine therapy is ongoing, evaluating ctDNA at baseline and over time (NCT04768426). One of the aims of this study is to inform future clinical trials about the applicability of ctDNA results for tumor-alteration-adapted treatment as an eight-week post-neoadjuvant window before capecitabine therapy.

### 6.3. Longitudinal LB Analysis for MRD Detection

In contrast, MRD detection could also be performed over a longer time by means of serial blood sampling (Table 4). This approach might enhance the likelihood of MRD detection as the dynamics during adjuvant therapy/follow-up are mirrored.

#### 6.3.1. CTCs

Already in 2011, it was recognized that the persistent detection of CK19 mRNA positive CTCs during the first five years of BC follow-up increased the risk of late relapse [138].

#### 6.3.2. Multimodal LB

In 2023, a multimodal LB approach including CTC quantification, phenotypic, transcriptomic, and genomic profiling of CTCs as well as mutation and methylation profiling of cfDNA detected MRD in 18% (3/13) of early BC patients. In these three early BC patients, multimodal LB profiling identified a relapse at least four years earlier than metastases could clinically be detected [139].

#### 6.3.3. cfDNA

Utilizing cfDNA for longitudinal MRD detection typically requires patient-specific mutation assays [140]. The following summarizes three tumor-informed approaches: (1) Whole-exome sequencing (WES) from tumor tissue to identify somatic mutations and subsequently, personalized mutation sequencing panels targeting 16 SNPs or indels. In early BC patients, this approach reached a sensitivity of 89% for MRD detection with a lead time of up to 24 months (median 8.9 months) with a specificity of 100% with none of the non-relapsing patients being ctDNA-positive [141]. These results led to the coverage of Natera’s Signatera assays by MediCare (Lawerence, KS, USA) in February 2023 in the US for all early BC patients [142]. (2) WES from tumor tissue and subsequently, patient-specific digital droplet PCR (ddPCR) panels targeting around 50 mutations. Germline variants and variants developed by clonal hematopoiesis of undetermined potential (CHIP) were subtracted by peripheral blood mononuclear cell (PBMC) sequencing. Within one year after surgery, MRD was detected with 19% sensitivity and the median lead time from the first positive test to recurrence was 18.9 months (maximum 39.2 months) [143]. (3) WES on tumor tissue and for each patient, RaDaR assays. In high-risk HR-positive, HER2-negative BC patients with no evidence of recurrence five years after diagnosis, the serial blood analysis by patient-specific RaDaR assays identified all patients with distant metastatic recurrences (7.2%) with a median ctDNA lead time of 12.4 months. However, 2/8 patients with ctDNA-positive results had not had clinical recurrence at the last follow-up—potentially being false-positives. In total, 1.2% of patients had no MRD but local recurrence was identified—indicating a false-negative ctDNA result [144]. These findings led to the Medicare coverage of the RaDaR assays for HR-positive/HER2-negative BCs with no evidence of disease five years after diagnosis in the US in July 2023 [145]. The latter study underlines the necessity to improve the sensitivity of the analyses. Potential approaches to increase the sensitivity require an increased cfDNA input amount (by higher blood volumes, better extraction methods or methods without the need for cfDNA extraction), enhanced numbers of markers (multiple mutations, genome-wide analysis, methylation, or fragmentation analysis) and an improved background-to noise ratio by proper controls and computational tricks as outlined by M. Murtaza at the AACR 2023.

While MRD detection by blood analysis with a lead time to recurrence detection by current clinical routine was shown, the clinical utility of this testing can only be proven by clinical trials selecting patients with MRD-positive results and randomizing them into standard and therapy escalation arms. One clinical trial investigating the latter is the c-TRAK-TN trial (NCT03145961). WES was conducted on tumor tissue from early TNBC patients after neoadjuvant therapy who did not achieve a pCR and after surgery (*n* = 185) and tumor-informed ddPCR assays to follow one to two mutations per patient were designed (*n* = 171). MRD detection by ctDNA profiling was conducted every three months, up to 12 months in 161 patients. Patients with MRD-positive blood results (*n* = 44) were selected for either observation (*n* = 13) or intervention with pembrolizumab for one year (*n* = 32). In total, 72% (*n* = 23) of the patients in the intervention arm already showed detectable relapse by staging at the time point of a ctDNA-positive result and inclusion in the intervention group [146]. This implies that blood analysis here might not be used for MRD forecast but rather for recurrence identification as an alternative to radiologically staging patients after neoadjuvant therapy with no pCR.

In summary, liquid biopsies have prognostic value in the early BC setting and can be used to anticipate MRD (Table 4), but clinical utility for this blood-based monitoring and consequent therapy (de)escalation approach is pending. Due to the current search for alternative endpoints in clinical trials, the guidance document published by the FDA in 2022, which indicates that cfDNA dynamics under therapy in the early BC setting might be considered a valid endpoint in clinical trials in the future, is of special interest [147].

## 7. Liquid Biopsies for Prognostication in the Metastatic BC Setting

We should differentiate between the predicative and prognostic value of biomarkers [148]. Prognostic factors serve to prognosticate the probable further course of the disease. This prognosis can be influenced by therapy. In contrast, predictive factors serve to predict a probable therapy effect [148]. In the following section, the prognostic value of LB for therapy decisions in the metastatic setting is discussed. In Section 8, the predictive value of blood-based biomarkers is elucidated.

In contrast to the blood-based approaches for MRD detection in the early BC setting, blood-based strategies for prognostication in the metastatic BC setting are far away from being proven clinically relevant. This is mostly due to a lack of clinical trials showing therapy (de)escalation strategies for metastatic BC patients with blood-based results of prognostic value. Nevertheless, a number of papers presented in the following underscore the prognostic value of a wide variety of LB analyses (Table 5). Independent of these studies, we like to refer to the systematic review of Duque and colleagues, who summarize in tabular form studies that prove the prognostic value of LB in BC [149]. The authors mentioned low methodological quality of the primary articles and highlight the heterogeneity of techniques and blood analytes studied in the primary articles. In total, 52.7% of reviewed studies analyzed cfDNA, 17.6% CTCs and 12.16% both CTCs and cfDNA. Studies from China, Japan, US, Greece, UK and Germany accounted for over half of the primary articles [149].

### 7.1. Other Analytes

Besides the most popular LB analytes, circulating miR-200 family members (namely miR-200a, miR-200b, miR-141, and miR-429) were shown to significantly correlate with progression-free survival (PFS) in cases analyzed at baseline of the new line of systemic therapy in MBCs [150]. Within the EFECT trial in MBCs, evaluation of the thymidine kinase 1 (sTK1) in plasma sample at baseline revealed prognostic value [151]. The LAMP2 protein levels in red blood cells of MBCs were related to OS [152] and it was speculated that the increase in cfDNA triggered the expression of the DNA-sensing protein TLR9 on the surface of red blood cells [153], which in turn might result in the altered protein composition within the red blood cells in MBCs compared to healthy donors. A set of metastasis- and stemness-related mRNAs were higher expressed in EVs from BC patients with poor OS than in those patients with increased OS [154].

### 7.2. cfDNA

Duque and colleagues concluded from their systemic review that more than half of all primary articles evaluated cfDNA for prognostication in BC [149]. In Germany, ctDNA is not recommended as prognostic marker in MBC and the Oxford LOE was evaluated as 2a [148].

Methylation pattern on genome-wide scale [155] or of five specific genes [156] in cfDNA drawn at baseline was shown to correlate with OS in MBCs. The cfDNA methylation analysis on genome-wide scale was even prognostic in case evaluated at week four after therapy initiation and dynamics from baseline to four weeks were informative about OS as well [155]. Similarly, cfDNA measures (here copy number changes and consequently, the genomic instability score) at baseline, after one week and two weeks after treatment initiation in MBCs were significantly associated with poor OS [157].

Tumor-derived cfDNA fractions can be identified by mFAST-SeqS z-scores and ichorCNA algorithms and results of both techniques associated with clinical outcome (PFS and OS) in small MBC cohorts [158]. In the BEECH trial investigating the benefit of the AKT inhibitor capivasertib in addition to paclitaxel in ER-positive advanced BCs, evaluation of ctDNA abundance after four weeks of therapy by mutation-specific ddPCR assays revealed a significant correlation with PFS independent of the therapy arm [159]. In addition, a higher number of pathogenic or likely pathogenic variants in the cfDNA is associated with worse OS [160]. In this regard, it was also described that a decrease in the mean variant allele frequency of cfDNA mutations in any of the 425 genes sequenced from baseline to cycle two in advanced BC patients treated with ICI was related to a longer PFS [161].

Mutations detected in cfDNA within specific genes were found to be associated with worse OS in MBC patients. In 2017, cfDNA *ESR1* mutations were found to indicate worse OS in a cohort of 42 pretreated MBCs and these mutations were more specifically associated with a shorter duration of endocrine treatment effectiveness in MBCs [162]. Evidence regarding the influence of endocrine treatment on the development of *ESR1* mutations increased over time and currently, knowledge of a therapy switch to estrogen receptor degrader in *ESR1* mutant patients is translating into clinical practice [163] (Section 8). *TP53* and/or *PIK3CA* mutations detected in cfDNA of MBCs were shown to indicate worse OS [164], even in small cohort studies [165]. In addition, a specific *BRCA1* mutation detected in the cfDNA of a stringent HR-positive/HER2-negative MBC cohort of 44 patients was associated with worse OS [160].

### 7.3. CTCs

With regard to the prognostic value of CTCs in MBC, the first study was published in 2004. Blood was drawn from 177 MBC patients before therapy initiation of any therapy in any therapy line and CTCs were quantified by the CellSearch system. The CellSearch system captures cells based on the surface expression of the epithelial marker EpCAM and cells are identified as CTCs when stained positive for Cytokeratin (CK) and negative for CD45. In a training cohort, a cut-off of five CTCs in 7.5 mL blood identified by CellSearch was shown to differentiate patients with good versus worse PFS and the CTC quantification with this cut-off correlated significantly with OS [166]. Multivariate Cox proportional-hazards regression showed CTC quantity at baseline to be the most significant predictor of PFS and OS. One year later, results from 83 patients within the same cohort were published [167]. Here only patients with newly diagnosed, measurable MBC who were about to start their first line of systemic therapy were included, the cohort was not separated into training and validation cohorts and the cut-off of five CTCs per 7.5 ml blood was applied. In total, 52% of patients had ≥five CTCs and these patients had a decreased PFS and OS compared to the patients with no or less than five CTCs. Ten years after the first study showing the prognostic value of CTCs in MBCs, a meta-analysis including 1944 MBC patients validated the significant association of CTC quantity (isolation via CellSeach; cut-off: 5 CTCs) regarding PFS and OS [168]. CTC count improved the prognostication of MBCs when added to the clinico-pathological predictive models. A variety of other studies exist that confirm the prognostic value of CTC numbers by CellSearch—even in more specific sub-cohorts like MBCs receiving first-line chemotherapy [169] or TNBC patients [170]. Additionally, it was reported that the presence of CTC clusters has additional prognostic value when compared to the single CTC quantification alone [171]. The number of CTCs within the clusters might also be related to OS [171]. The prognostic value of CTC clusters was also identified in first-line treated MBCs [172].

The knowledge of epithelial–mesenchymal transition (EMT) and detection of CTCs with EMT-like features [173] opened up the search for further selection technologies to detect these cells. In 2009, it was shown that the CTC presence was significant associated with PFS [174] in MBC patients in case the selection of CTCs was based on the surface protein markers EpCAM and MUC1 and detection of CTCs was based on HER2, MUC1 and GA733-2 transcript expression. Ten years later, it was reported that MBC patients can be stratified into patients with good versus worse PFS and OS based on a more detailed molecular characterization of their CTCs [175]. In this study, CTC isolation was based on the negative immunomagnetic selection by RosetteSep^TM^ CTC Enrichment Cocktail, containing a CD56 antibody. The transcript expression of PALB2, MYC, EpCAM, VIM and ALDH1, besides others, was quantified in the fraction of enriched blood cells. MBC patients with CTCs showing high PALB2 or MYC transcript expression had a shorter PFS and OS [175]. In addition, patients with CTCs showing epithelial-stem cell like features (EpCAM^high^VIM^low^ALDH1A1^high^ signature) also showed shorter PFS and OS [175]. In a specific cohort of MBC patients before eribulin treatment, CTC isolation by a microfluidic chip and characterization by vimentin (mesenchymal marker) and pan-cytokeratin (CK; epithelial marker) staining was used [176]. The entirety of epithelial and mesenchymal CTCs, as well as only the epithelial or mesenchymal CTCs was related to OS [176]. Another example for the prognostic value of CTCs in MBCs determined by not only the epithelial marker EpCAM, but also by markers for the EMT-feature and stem cell feature was presented in 2021 [177]. While CTC isolation was based on cell density and EpCAM protein expression on the surface, the molecular characterization included a multimarker quantitative PCR (qPCR), targeting stem cell markers (CD24, CD44, ALDH1), the epithelial marker CK-19, the mesenchymal marker TWIST and BC-specific markers (ESR1, PR, HER2, EGFR). The univariate Cox regression model showed prognostic value for the presence of CTCs with either CK-19 overexpression, HER2 overexpression or CTCs with CD44high/CD24low or ALDH1high/CD24low features [177].

However, properties independent of epithelial, mesenchymal and stem cell features may also show prognostic value in CTCs of MBCs. In this regard, HER2-negative MBC patients showed reduced OS in case CTCs with strong HER2 staining were detectable [178], potentially showing the temporal and/or spatial heterogeneity of the tumor cells. Additionally, characterization of CTCs with regard to their mitotic activity increased the hazard ratio for association with OS in MBCs dramatically compared to CTC quantification itself [179]. With regard to specific therapy, it is important to note that a-8-gene predictor (EEF1A, PTRF, CXCL14, ERBB3, EGFR, PTPRK, KRT81, TWIST1) was published to be related to PFS in first-line aromatase inhibitor (AI) treated patients, while the same predictor was not related to PFS in MBCs treated with other therapy regimens [180]. In conclusion, although CTC quantification for prognosis is FDA-approved and recommended by the German work group for gynecologic oncology [148], but a treatment decision outside of clinical trials based on CTC numbers or phenotypes is explicitly discouraged [148].

It was independently shown that molecular characterization of CTC and the CTC identification with markers other than the epithelial marker EpCAM might be even more relevant for prognostication in MBCs [174,175,176,177,178,179,180].

### 7.4. Multimodal LB

Shaw et al. demonstrated that CTC counts by CellSearch and also total cfDNA level were associated with OS in MBCs and thus, concluded CTCs and cfDNA to be equally valuable OS markers [35]. A more concrete analysis of a study conducted by Fernandez-Garcia et al. showed that both, the total cfDNA amount and the number of CTCs were related to OS, however, the combined analysis of CTCs and cfDNA was more informative regarding OS than the sole analysis of one of the analytes [181]. Similarly, Ye et al. concluded CTC counts and cfDNA levels to forecast OS in sole analysis, but in joint analysis the association with OS was stronger [182]. An additive value of CTC and cfDNA analysis to forecast OS was further published by Bortolini Silverira et al. [88], showing that CTC counts by CellSearch and ctDNA identified by targeted NGS had non-overlapping profiles and correlated in sole and also in combined analysis with OS in the UCBG COMET study (NCT01745757) that applied first-line paclitaxel and bevacizumab [88].

Using molecular characterization for both, CTCs and cfDNA, Keup et al. found ESR1 variants in CTCs and cfDNA to indicate worse OS [183], while Chimonidou et al. evaluated the SOX17 promotor methylation in cfDNA and CTCs to be of prognostic relevance [184]. Evaluating more than one analyte from the same blood sample showed the prognostic value of both, CTCs (CK-positive or no CK but with HER2-positive staining) and EVs (CK-positive or no CK but with HER2-positive staining) [185]. The potential of a combination of CTC and EV results for OS prognostication was not stated. However, the heterogeneity of CTCs or EVs within one blood sample was shown to be inversely associated with OS [185]. One further study combined the analysis of CTCs and of disseminated tumor cells (DTCs) in the bone marrow [186] and concluded that DTCs as well as CTCs were significantly associated with worse OS, but to what extend a combination of both analyzes might improve OS prognostication was not documented. However, it was stated that there was no significant association of DTC and CTC results, potentially representing different aspect of systemic BC spread [186]. A multimodal LB approach in MBC patients was conducted by Keup et al. [10]. In the ELIMA project, the value of cfDNA, CTC genomic DNA, CTC mRNA and EV mRNA for—among others—prognostication was elucidated in MBC patients. The transcriptional and genomic evaluation of all four analytes in only 20 mL of blood revealed their additive value for prognostication, as the so-called ‘ELIMA.score’ showed a significant correlation with OS with a decreased *p*-value when compared to each single analyte [10,187].

## 8. Liquid Biopsies for Therapy Guidance in Breast Cancer Management

Liquid biopsies also harbor a huge potential for therapy guidance. In general, the molecular characterization of tumor-derived components or tumor-associated components in the blood can shed light on potential therapy targets or can otherwise guide the therapy selection. We have to distinguish between actionable targets, biomarkers for resistance and biomarkers for adverse events, all of which are suitable to guide therapy selection. Regarding biomarkers for resistance, only markers for de novo resistance allow for therapy selection, while markers for acquired resistance are relevant for therapy monitoring (Section 9).

As blood-based biomarkers for adverse events under therapy, levels of specific circulating miRNAs and cfDNA methylation were suggested [188,189]. Both analytes were described to relate to cardiotoxicity either in early TNBC patients under epirubicin/cyclophosphamide-docetaxel neoadjuvant chemotherapy [188] or in HER2-positive BC patients who received anthracycline-based chemotherapy [189].

The majority of LB markers for therapy guidance are mutations analyzed in either cfDNA or CTCs. Based on the frequently asked question of whether cfDNA mutations mirror the mutational status of the tumor tissue, it is concluded that the time interval between tissue biopsy and blood draw influences the concordance. In a study in which the tissue biopsy and blood draw were at maximum within a time frame of 12 weeks, the mutation concordance was reported to be 79–91%, depending on the gene analyzed [190]. An earlier study included 45 BC patients and reported an agreement of 91–94.2% for wildtype and mutant alleles, but in case only the mutations were considered, the reported concordance was 10.8–15.1% [191]. This study, conducted in 2016, reported that a higher variant allele frequency in the cfDNA was associated with a higher concordance [191], which implies that the sensitivity of the mutation detection in the cfDNA was an issue back in the earlier years when methods were first established. Regarding the concordance of mutation detection in CTCs versus tissue in MBCs, it was described that 95% concordance existed in at least one mutation or copy number variant alteration and in some patients, actionable alterations were found either in tissue or in CTCs [192]. While in specific cases, the mutational analysis of cfDNA recently got into clinical practice for MBCs, lack of standardization of (pre-)analytical workflows, heterogeneous study results, the lack of appropriate indications and timing of blood draw and difficulties with result interpretation limit the usage of LB in clinical practice at the moment [193].

Although the DESTINY-Breast04 trial (NCT03734029) trial showed that not only HER2-positive patients benefit from Trastuzumab Deruxtecan therapy, which targets anti-HER2, but also patients with very low levels of HER2 signals (previously defined as HER2-negative; now defined as HER2low) [194], in general, the inclusion of biomarkers into clinical trial designs got essential and subgroup analysis mostly identified a biomarker specific cohort to show the greatest benefit. Additionally, biomarker-driven trials exist that include either patients based on the presence of a specific biomarker (basket trial) or that include patients who share the same cancer histology, but are allocated to different arms, based on their biomarker status (umbrella trial) [195].

One of the first umbrella trials utilizing cfDNA mutation analysis in MBCs was the plasmaMATCH trial [196]. Patients were allocated into four different treatment arms depending on their ctDNA mutations. In cohort A, patients with ESR1 mutations received Fulvestrant, a selective ER degrader (SERD). In Cohort B, patients with HER2 mutations were treated with Neratinib (plus Fulvestrant for ER-positive patients). Cohort C, ER-positive patients with AKT1 mutations were treated with Fulvestrant and Capivasertib, an AKT inhibitor and cohort D consisted of ER-negative patients with AKT1 mutations and patients with PTEN mutations who received only Capivasertib. In cohorts B and C, the target number of responses (metastases diameter stable or decreased) was met or exceeded while treatment with Fulvestrant alone in ESR1 mutant patients did not reach the target number of responses. Treatment with Capivasertib alone in ER-negative patients with AKT1 mutations and patients with PTEN mutations was not effective in this trial [196]. The investigators reported expert methodology, well-written protocols and early engagement with regulators as one of the keys to implementing the plasmaMATCH trial [197].

In the following, the advantages of multimodal LB evaluations, the opportunity of LB analysis for the advancement of the molecular tumor boards and published LB approaches for the guidance of specific therapies are elucidated.

Mutation profiling is the major analysis for therapy guidance in BCs, at the moment. It was shown that the mutational profiling of CTCs and cfDNA from the same blood sample has additive value to identify actionable mutations [183]. The basis for this evaluation was performed by a comparison of cfDNA mutations isolated from whole blood compared to matched CTC-depleted blood [198]. Neither the number of detected variants per patient nor the number of exclusively detected variants per patient in only one cfDNA source nor the characteristics of the exclusively detected cfDNA variants differed between the two matched cfDNA sources. Thus, parallel isolation of cfDNA and CTCs from only 10 ml of blood in an “all from one tube” format was feasible [198]. Exactly this approach was used in a small cohort of HR-positive/HER2-negative MBC patients and revealed *BRCA1* and *BRCA2* variants to be more frequently detected in CTCgDNA than in cfDNA. In contrast, *PIK3CA* and *ESR1* mutations were more common in cfDNA compared to CTCgDNA. Thus, mutational analysis of both analytes maximizes the number of patients with actionable mutations [183]. Even more blood analytes were evaluated in the so-called ELIMA project analyzing cfDNA, CTC gDNA, CTC mRNA and EV mRNA from only 18 ml of blood. Each analyte added information that was absent in the other ones and the multimodal approach resulted in a maximum number of patients with actionable signals, showing the complementary nature of the analytes for therapy guidance [10,187].

In recent years, knowledge about the genomic landscape, especially MBCs, has increased dramatically using NGS for direct comparison of primary tumor and metastasis pairs [199,200,201]. The genomic landscape of MBCs in cfDNA was also recently reported [160,190,202,203,204,205]. After standard treatment options are exhausted, inclusion into a molecular tumor board and genomic profiling of the tumor/plasma may enable the detection of actionable variants. Difficulties in interpreting genomic data for therapy guidance were addressed by a publication comparing the output (treatment recommendations) of different commercial clinical decision support tools using the same data input [206]. Each platform was based on different variant classifications, which resulted in major discrepancies between the treatment recommendations at the end [206]. To pick only the genomic alterations from the genomic landscape of BCs that have an LOE for actionability, the ESMO defined the ESCAT (ESMO Scale for Clinical Actionability of Molecular Targets) scale [207,208]. In 2019, the level of evidence (LOE) I was rewarded for ERBB2 amplifications, BRCA1/2 mutations and PIK3CA mutations, microsatellite instability (MSI) and NTRK translocations [208], while ESR1, ERBB2 and AKT1 mutations, as well as PTEN loss, were rated with LOE II. The platform OncoKB also defined the LOE of genomic alterations [209], among others based on FDA LOE and the variant classification of the AMP/ASCO/CAP Consensus Recommendation [210]. With reference to the OncoKB scale of LOE, it was reported that matched plasma and tissue sequencing of BC patients revealed 54% of patients having concordant actionable variants detected in plasma and tissue, while more patients (29%) showed an actionable variant only in the tissue compared to only in the plasma (20%) [211]. Most of the variants detected only in the tissue were rated OncoKB LOE I/II (72%). However, in the longitudinal setting of luminal BC patients, more variants were only detected in the plasma, mainly emerging *ESR1* mutations. Referring to the ESCAT scale, 1500 MBC patients were screened for mutations in the SAFIR02-BREAST trial, but in only 150 patients a variant rated with ESCAT LOE I was detected [212]. In half of these 150 patients, a BRCA mutation was detected. Targeted therapy matched to the genomic alterations improved the PFS only in case the variants were ESCAT LOE I or II [212]. The definition of actionable variants by other means resulted in reports of 44–95% of patients having at least one actionable variant for therapy guidance [213,214,215,216,217,218,219]. Only in 40/104 patients with actionable alteration, clinical management was changed [219]. The 1-year OS rate was 65.9% in women treated based on their genomic alteration versus 22.7% in patients who received standard chemotherapy [218].

### 8.1. Chemotherapy (CTX)

Decision-making for/against chemotherapy regimens in the early BC setting has been revolutionized by the TAILORx trial and the OncoType Dx test [220]. However, the latter one is conducted with tumor tissue and the translation of this gene expression profiling from tissue to blood components might be possible, but has not been published in any regard yet. Here, we like to refer to the sections’ prognostication and therapy escalation in the early and metastatic BC setting (Section 4, Section 5, Section 6 and Section 7), as biomarkers for worse prognosis that might in the future be useable to select therapy escalation strategies.

The translation from prognostic value to value for therapy guidance was approached in the STIC CTC trial with regard to CTC quantification in MBCs [221] (Table 6). First-line therapy selection in HER2-negative MBCs was conducted either based on the CTC quantity by CellSearch where patients with <5 CTCs per 7.5 mL blood were treated with endocrine therapy, while those with ≥5 CTCs with chemotherapy or by clinicians’ choice without knowing the CTC counts. In general, PFS and OS were equally distributed in all groups, however, in patients with no concordant stratification status (high risk by clinicians/low CTC number or low risk by clinicians/high CTC number), chemotherapy prolonged PFS and OS compared to endocrine therapy [221].

Ubiquitin carboxyl-terminal hydrolase-L1 protein levels in EVs [222], regulating the drug resistance-associated P-glycoprotein, as well as circulating miR-125b were shown to predict response to CTX before therapy initiation [223]. Acquired chemo-resistance was associated with levels of the transient receptor potential channel 5 (TrpC5) in EVs [224,225], not usable for therapy guidance but therapy monitoring.

### 8.2. PARP Inhibition

In total, 60–70% of persons with germline (g) BRCA1/2 mutations develop BC [226] and genetic predisposition accounts for 5–10% of all BC cases [227]. In total, 2.6% of BC patients have germline *BRCA1*, *BRCA2* or *PALB2* mutations [228] and 1.7% of all BC patients have germline *CHEK2* or *ATM* mutations [228]. In total, 11% of all TNBC patients present with gBRCA1/2 mutations [229]. Patients with ER-positive tumors and gBRCA1/2 mutations have a 2.3 higher risk of recurrence and 3.4 higher risk of death compared to patients with ER-positive tumors but no gBRCA1/2 mutation [230]. Additionally, it was shown recently that patients with gBRCA1/2 mutation and ER-positive tumors had worse outcomes compared to patients with gBRCA1/2 mutation but ER-negative tumor [230], which might be explained by the current clinical practice in testing for gBRCA1/2 mutations and selecting therapy regimens as further described.

**Figure 2:**
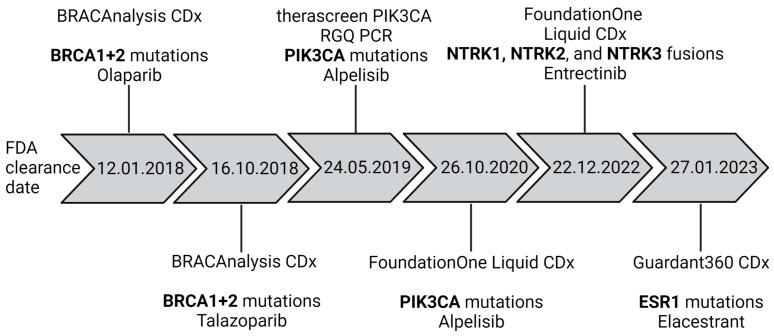
Liquid Biopsies for therapy management of BC patients. Different liquid biopsy analytes were evaluated for their suitability for clinical applications over the entire course of the BC disease. The references studying the specific blood analytes for the specific clinical BC application are listed. Created with BioRender.com.

In early BC, gBRCA1/2 mutations are of prognostic value to achieve a pCR under chemotherapy and forecast DFS under PARP inhibition in HER2-negative patients [98]. The PARP inhibitor Olaparib is recommended for early TNBC patients showing no pCR and harboring gBRCA1/2 mutations as well as for high-risk gBRCA1/2 mutant HR-positive/HER2-negative early BC patients as proven in the OLYMPIA trial [98,231] (Table 7). Thus, it is a standard to test HER2-negative BC patients for gBRCA1/2 mutations (ESCAT scale IA) [98,208]. Germline mutations can easily be detected in all body cells, thus, BRCA1/2 mutation testing was also approved by the FDA in whole blood (BRCAAnalysis CDx) (Figure 2).

The OlympiAD trial resulted in approval of Olaparib therapy and the EMBRACA trial in the approval of Talazoparib, another PARP inhibitor, in pre-treated MBC patients with gBRCA1/2 mutations [6,148,232,233,234]. In the case of platinum-based-therapy naïve patients, carboplatin is recommended in gBRCA1/2 MBC carriers in contrast to Docetaxel [6,148]. While HR-positive/HER2-negative gBRCA1/2 mutant MBC patients in Germany currently receive PARP inhibitors earliest in second-line regimens after CDK4/6 inhibition, metastatic TNBC patients might receive PARP inhibition in any therapy line depending on their PD(L)1 status (expression of programmed cell death protein-1 and/or its ligand) and gBRCA1/2 status [148]. The Olaparib Expanded trial also showed the effectiveness of Olaparib in MBC patients with germline PALB2 or in MBC patients with somatic BRCA1/2 mutations [235], while MBC patients with germline mutations in the HRD-associated genes CHEK2 and ATM did not benefit from Olaparib treatment. At the moment, Olaparib therapy is rated as an option for MBC patients with somatic BRCA1/2 mutations (ESCAT scale IIA) or germline PALB2 mutations (ESCAT scale IIA) by the ESMO guideline and is rated with +/− by the German AGO [6,148].

### 8.3. Anti-HER2 Therapy

The potential of LB analysis for therapy selection of anti-HER2 treatment can be stratified into the analysis of HER2 expression on the RNA or protein level in/on CTCs, ERBB2 amplification as well as mutation evaluation in cfDNA.

In general, the HER2 protein expression status in the tumor tissue was found highly dynamic under therapy in the Penelope-B trial [236]. It was further reported that HER2 status evaluation, currently used in clinical practice might not reflect the potential benefit of anti-HER2 therapy, as single HER2-positive tumor cells were not sufficient to classify the tumor asHER2-positive [194,237]. The benefit of anti-HER2 therapy in HER2-negative patients has already been reported in 2008 [238], and recently confirmed in a large clinical trial [194] changing the HER2 classification procedure [239]. In 2010, it was reported that HER2 protein expressing CTCs can be detected in the blood of HER2-negative MBC patients [240,241,242]. As underlying mechanisms, the inadequate HER2 evaluation of the tumor tissue and consequently, the presence of HER2-positive cells in the tumor tissue and their extravasation into the circulation, as well as adaptation processes during extravasation inducing HER2 expression were suggested. Treatment with trastuzumab prolonged the DFS in HER2-negative early BC patients with CK19-positive CTCs present before and after adjuvant chemotherapy compared to observation [243] (Table 8). More precisely, 89% (51/57) of the patients had CK19-positive ERBB2-positive CTCs at baseline. The fraction of patients with CK19-positive CTCs after trastuzumab treatment was reduced to 14%, while observation led to 17.9% of patients with CK19-positive CTCs. These results led to the hypothesis that HER2-negative patients with ERBB2-positive CTCs could achieve a better outcome by applying anti-HER2 therapy with a CTC-based therapy selection [244]. To prove this hypothesis, two trials were conducted. However, neither the treatment with lapatinib of pre-treated HER2-negative MBC patients with HER2-positive or EGFR-positive CTCs [245,246] nor the treatment with TDM-1 of pre-treated HER2-negative MBCs with ERBB2 amplified CTCs in the CirCe T-DM1 trial [247] resulted in an objective benefit. Results of the DETECT III trial (NCT01619111) are still pending and might shed light on how CTC isolation and analysis as well as treatment regimen should (not) be designed to still keep up the hope for anti-HER2 treatment in HER2-negative patients harboring HER2-positive CTCs in the future. As one opportunity for further trials, it is important to note that the levels of HER2-positive EVs in the circulation match the number of HER2-positive tumor cells in the tissue [248].

In contrast, cfDNA ERBB2 mutation analysis has the potential to be integrated into clinical practice in the future. Some ERBB2 mutations result in a truncated HER2 isoform which cannot bind to lapatinib, indicating lapatinib resistance. However, neratinib is able to bind the truncated form of HER2. This might explain why several MBC patients with ERBB2 mutations were resistant to lapatinib, but sensitive to neratinib [249,250,251]. ERBB2 mutations were most frequently detected in lobular BCs [252]. Cohort B in the plasmaMATCH trial also showed a benefit of neratinib treatment in ERBB2 mutant MBC patients [196]. However, outside of clinical trials, the ERBB2 mutation testing in tissue or plasma is not recommended and neratinib is not approved for ERBB2 mutant BCs (ESCAT scale IIB) [148,208].

In addition to ERBB2 mutations analysis, ERBB2 CNV analysis should be mentioned. Genomic profiling of cfDNA alterations in MBCs revealed 96.4% of all patients with ERBB2 CNVs to be HER2-positive patients and among the HER2-positive MBC patients, the most frequently altered genes were TP53, PIK3CA and ERBB2 [190]. The CNV analysis of ERBB2 in cfDNA from MBC patients however has never been reported for therapy selection.

### 8.4. PIK3CA Inhibition

PIK3CA mutations were detected in BC patients for the first time in 2004 [253]. PIK3CA mutations were detected in 20–30% of all BC patients [254,255] and thus, range among the most commonly mutated genes (among TP53 and ESR1) in BC patients [190]. In luminal MBC patients, the PIK3CA mutation prevalence was reported to be 40% [256]. PIK3CA H1047, E545K and E542K account for 70–80% of all PIK3CA mutations in BC [226], so the mutations are located in specific hotspots of the gene. PIK3CA mutations were frequently studied in the plasma. The prevalence of PIK3CA mutations detected in cfDNA of MBCs was 43.3% in the BOLERO-2 trial [257] and 33% in the PALOMA-3 trial [258]. In early BC patients, a prevalence of 22% by dPCR in cfDNA was reported and rarely, different PIK3CA mutations occurred simultaneously in the same sample [259]. From 2012 to 2015, comparisons of PIK3CA mutation detection in tumor tissue and plasma revealed a concordance of only 27.5% [260] to 72.5% [261]. In 2018, a concordance of 83% was reported for PIK3CA mutation detection in metastatic tissue and cfDNA using ddPCR [262], indicating the importance of sensitive cfDNA mutation detection.

To add a dimension of complexity, PIK3CA mutations were also detected in CTCs. In total, 16% to 33% of all MBCs were reported to harbor PIK3CA mutant CTCs [263,264]. And the PIK3CA mutational status was found concordant in cfDNA and CTCs isolated from the same sample from MBC patients [265]. In a case study, PIK3CA mutant CTCs were detected in a longitudinal sampling of two patients with emerging endocrine resistance [192].

The BELLE-2 trial was the first to determine the utility of a PI3K inhibitor (Table 9), here buparlisib, in cfDNA PIK3CA mutant and wildtype HR-positive/HER2-negative MBC patients progressing under aromatase inhibitor (AI) therapy [164,266]. Mutation analysis in the plasma was conducted by BEAMing and a concordance of 77% between the PIK3CA mutation status of tissue and plasma samples was reported. Among all patients, 21% showed PIK3CA mutant cfDNA and the addition of buparlisib improved the PFS in PIK3CA mutant HR-positive/HER2-negative MBC patients progressing under AI, but also significantly increased the adverse events [164,266]. Using the same PI3K inhibitor, the BELLE-3 trial demonstrated a benefit in patients with prior use of an mTOR inhibitor; however, here it was independent of the PIK3CA status in ctDNA [267].

To protect patients from side effects, the results of the SOLAR-1 study led to the approval of another PI3K inhibitor in the EU in 2020, alpesilib, for PIK3CA mutant HR-positive/HER2-negative MBC patients [268]. In the SOLAR-1 study, worse PFS of PIK3CA mutant MBC patients was improved by the application of alpesilib to an extent of a PFS achieved in PIK3CA wildtype MBC patients, that did not benefit from alpesilib treatment. As the first FDA-approved assay for guiding treatment decisions in BC using plasma specimens as an LB, the therascreen^®^ PIK3CA RGQ PCR Kit was used as a companion diagnostic (CDx) for alpesilib treatment, to detect PIK3CA mutations in cfDNA or tissue by qPCR (FDA approval P190001 and P190004) (Figure 2). Subsequently, the FDA also approved the FoundationOne Liquid CDx test for PIK3CA mutation detection (Figure 2).

Alpelisib in combination with fulvestrant for PIK3CA mutant HR-positive/HER2-negative MBC patients after progression under AI is recommended by the ESMO [6,256] stating that ctDNA assessment for PIK3CA mutation analysis is an option besides mutational profiling in tissue samples. In patients with no available archival tumor tissue, ctDNA assessment is recommended [256]. PIK3CA mutations are classified as tier IA by the ESMOs’ ESCAT scale [6,208]. In the US, the ASCO (American Society of Clinical Oncology) also recommends alpesilib with the additional statement that PIK3CA mutations can be detected in tumor tissue or cfDNA, but in case of a negative cfDNA result, tumor biopsy should be considered due to the higher variant allele frequency of potential mutations [269].

The report of a missing significant OS benefiting from alpesilib treatment [270] resulted in an interruption of reimbursement for alpesilib in France and alpesilib was withdrawn from the market in Germany on the first of May 2021 by Novartis. Updated in 2022, the current ASCO guidelines recommend alpesilib [271] in PIK3CA mutant cases. The ESMO rated PIK3CA mutation analysis with ESCAT scale IA [6] and in Germany, the recommendation for PIK3CA mutation profiling in primary tumor tissue, metastasis or plasma was confirmed in 2023 [148]. Meanwhile, CDK4/6 inhibitors were approved for HR-positive/HER2-negative MBC patients and the BYLieve trial showed the efficiency of Alpesilib plus Fulvestant after progression under CDK4/6 inhibition [272]. Consequently, Alpesilib is used earliest in second-line treatment of MBC patients [148].

In the future, alpesilib might be applied in more HER2-negative patients because its application (NCT02379247) demonstrated that in combination with nab-paclitaxel, a prolonged PFS could be achieved in heavily pre-treated patients with PIK3CA mutation in tumor or plasma compared to PIK3CA wildtype patients [273].

### 8.5. Endocrine Therapy (ET)

In early BC, mutations in the ER gene (ESR1) cannot be found in tumor tissue [274,275]. However, after endocrine treatment, *ESR1*, *ERBB2*, *NF1*, *EGFR* and *MYC* variants were significantly increased in FFPE tissue of HR-positive BC patients in the MSK-IMPACT study [200] with ESR1 variants presenting in a prevalence of 20–50% [275,276]. The prevalence was dependent on the number of received treatment lines [277] as well as the type of endocrine treatment [278]. ESR1 mutations in advanced BC patients have already been described in 2013 [274,279,280,281,282] with most mutations occurring in the ligand-binding domain, resulting in a ligand-independent and constitutively activated receptor associated with aggressive disease biology [278].

In 2015, ESR1 mutations were described to be found in cfDNA in MBCs for the first time [283]. Prior use of aromatase inhibitor (AI) treatment was significantly associated with the prevalence of ESR1 mutations in cfDNA [190]. With a prevalence of 25–40%, ESR1 variants are among the most common variants in the plasma of HR-positive MBCs, along with PIK3CA and TP53 variants [162,190]. A longitudinal cfDNA mutation analysis has further shown that ESR1 variants emerged under afinitor and aromasin treatment and were detectable eight months before progression was identified by radiographic staging [160]. A comparison of ESR1 variant detection in tissue and plasma in 171 advanced BCs by dPCR revealed 97% concordance with 75% sensitivity and 100% specificity for ctDNA analysis compared to tumor DNA [282].

In addition to cfDNA, ESR1 variants were also described in CTCs using the CellSearch platform, followed by single-cell isolation via DepArray technology [284]. More specifically, ESR1 D538G and an ESR1 single copy loss were described in single CTCs of an endocrine-resistant MBC [192]. A direct comparison of ESR1 variant detection by ddPCR in matched cfDNA and CTCs of MBC patients revealed the allele frequency in cfDNA to be greater than in CTCs [285], probably caused by a high leukocyte contamination in the CTC fraction [286]. Additionally, the sensitivity of ESR1 variant detection was higher in cfDNA than in CTCs [285]. There is a question of whether these results hold true in case other CTC isolation methods and variant detection methods are used.

From the clinical perspective, it is important to note that 40% of all patients receiving first-line endocrine therapy will not benefit [287], 40% of all patients receiving tamoxifen will finally relapse [288] and all MBC patients receiving endocrine therapy will eventually develop resistance [287]. To explain the impact of these therapies, the mechanism of action of tamoxifen, AI and SERDs in ESR1 mutant patients should be taken into account [289]: (1) Tamoxifen binds to ER and inhibits dimerization. Consequently, ER cannot work as a transcription factor and gene expression resulting in cell growth and proliferation is inhibited. (2) AIs inhibit the aromatase enzyme, converting androgen to estrogen. Thus, the concentration of estrogen is reduced and less ligands to bind to the ER are available, resulting in less ER proteins that function as transcription factors for the expression of proteins important for cell growth and proliferation. (3) Selective ER degrader (SERDs) result in the degradation of the ER itself. Reduced ER concentration causes less expression of proteins relevant for cell proliferation. Keeping these mechanisms of action in mind, it is to conclude that tamoxifen and AI will not inhibit the function of mutated ER, while SERDs lead to the degradation of the entire mutated constitutively active ER protein and can reduce cell proliferation.

With this knowledge, it is not surprising that ER mutations or ER fusions were shown to cause resistance to (specific types of) endocrine therapy [162,274,279,280,281].

In this context, the plasmaMATCH trial, treating MBC patients with ESR1 mutations in the cfDNA with fulvestrant demonstrated that only 8% of patients (6/74) responded, questioning the effectiveness of fulvestrant in this patient group [196].

In contrast, direct comparison of fulvestrant (SERD) with exemestane (AI) in the SoFEA trial, showed a significantly prolonged PFS using fulvestrant compared to exemestane in ESR1 mutant HR-positive MBC patients [290] (Table 10). The combined analysis of data from the SoFEA and EFECT trial even showed an OS benefit for ESR1 mutant MBC patients treated with fulvestrant compared to exemestane [291]. The PADA-1 trial showed the benefit of a switch from AI to fulvestrant after progression under AI and detection of ESR1 mutations in the plasma to result in a PFS of 3.5 months [276]. However, longitudinal monitoring via ESR1 mutation detection in the plasma under AI treatment and switch to fulvestrant plus CDK4/6i compared to continuation of AI after emergence of ESR1 mutations without radiographic evidence for progression increased the PFS from 5.7 months to 11.9 months [276].

Elacestrant, an new oral SERD, was recently shown in the EMERALD trial to significantly increase the PFS of ER-positive/HER2-negative MBC in the second or more therapy line after progression under CDK4/6i and one previous chemotherapy line at maximum compared to standard endocrine monotherapy [163]. This effect was shown for both, ESR1 mutant and ESR1 wild-type patients. The hazard ratio however, showed a greater effect of PFS prolongation from elacestrant compared to standard endocrine monotherapy or specifically to fulvestrant in ESR1 mutant patients compared to all patients, independent of their ESR1 status. The adverse effects were manageable and in contrast to fulvestrant, elacestrant can be taken orally.

The SoFEA trial data did not result in the recommendation of ESR1 mutation detection for therapy decision against AI by the ESMO and in 2020, ESR1 mutations were rated with the ESCAT scale tier IIA [256]. However, evidence for ESR1 mutation detection increased in- and outside the EMERALD study, resulting in the FDA approval of the Guardant360 CDx test for ESR1 mutation detection in codons 310–547 in cfDNA as companion diagnostics (CDx) for treatment with elacestrant (Figure 2 and Table 15). Consequently, the ASCO published an update on the ESR1 mutation detection recommendations [292]. Testing for the emergence of ESR1 mutations is now recommended in ER-positive/HER2-negative MBCs at the time of recurrence or progression on endocrine therapy. Blood-based ESR1 mutation detection is preferred over tumor tissue testing due to the higher sensitivity [196]. In patients with ESR1 wild-type results in blood and tissue, re-testing should be performed at the subsequent progression(s). In HR-positive/HER2-negative MBC patients with prior CDK4/6i therapy and presence of ESR1 mutation in blood or tissue, elacestrant is recommended by the ASCO [292]. Data showing benefit of a combination of elacestrant with targeted agents are, however, still missing. In Germany, the update of the AGO guidelines in 2023 included ESR1 mutations as a parameter for therapy decision making [148]. At the time of publication of the AGO guidelines 2023, the EMA approval was still missing, but still the therapy of ESR1 mutant HR-positive/HER2-negative MBCs with elacestrant after CDK4/6i treatment for longer than 6–12 months was included in the therapy decision algorithm with LOE of 1b [148]. In September 2023, the EMA approved elacestrant in the European Union as treatment for postmenopausal women and men, with ER-positive, HER2-negative, locally advanced or metastatic breast cancer with an activating ESR1 mutation who have disease progression following at least one line of endocrine therapy including a CDK 4/6 inhibitor [293].

In the future, not only the ESR1 mutation detection but also methylation of the ESR1 promotor in cfDNA and CTCs might become relevant for selection of an endocrine therapy [294]. Interestingly, fusion of the ESR1 gene with other genes (like *AKAP12*, *ARMT1* and *CCDC170)* have been shown to occur even in early BC patients and as the ligand binding domain of ESR1 has been replaced, ER fusion proteins are constitutively active, similar to mutant ER proteins [295]. Despite ER signaling, evidence for the involvement of other signaling pathways (like ERBB2, PIK3CA, NFkB, FGFR1 and FOXM1 [196,296,297,298,299]) might increase and potentially become parameters for therapy decision making for/against the different endocrine therapy types.

In addition, other oral SERDs might find their way into clinical practice which might be used first-line and probably in combination with CDK4/6i. In this regard, camizestrant is under evaluation in a series of SERENA trials. The AMEERA-5 trial is ongoing using Amcenestrant, Giredestrant is evaluated in the PersevERA trial and Imlunestrant in the EMBER-3 trial. Additionally, the irreversible ER degrader PROTAC is applied in the Veritac trial and the oral selective ER modulator (SERM) lasofoxifen is evaluated in the ELAINE trials. It remains exciting whether ESR1 mutation detection as a predictive biomarker test gains relevance in the future to select SERDs for ESR1 mutant patients or whether endocrine therapy other than SERDs (like AI and tamoxifen) loses relevance and the only endocrine therapies in the market will be SERDs. In this case, ESR1 mutation testing would not be necessary.

### 8.6. AKT Inhibition

In a few BC patients (4% [300] and 1.4% [277]), AKT1 mutations were described with AKT1 E17K to be by far the most common variant in this gene [300]. Besides tissue analysis, AKT mutations were also described in cfDNA [277] and CTCs [301,302]. In AKT pathway-altered (*PIK3CA*, *AKT1*, or *PTEN)* HR-positive/HER2-negative advanced BC patients after progression under endocrine treatment, the CAPItelle 291 study showed a significant increase in PFS (7.3 months versus 3.1 months) using the AKT inhibitor capivasertib in combination with fulvestrant compared to placebo and fulvestrant [303]. However, the prevalence of patients with adverse events leading to discontinuation was 13% in the capivasertib arm compared to 2.3% in the placebo arm. In the plasmaMATCH trial (Table 11), ER-positive/HER2-negative MBC patients with AKT1 mutation in the cfDNA received capivasertib plus fulvestrant (cohort C) and this cohort met or exceeded the target number of responses with 4/18 patients [196]. However, 22% of patients showed fatigue with grade 3 to 4 and one patient in this cohort died by grade 4 dyspnea.

In metastatic TNBC patients, the addition of capivasertib to paclitaxel compared to paclitaxel alone correlated with a prolonged PFS and OS, especially in patients with *PIK3CA*, *AKT1* or *PTEN* alterations [304] in the PAKT trial. The most common grade ≥ 3 adverse events were diarrhea (13%), infection (4%), rash (4%), and fatigue (4%) in the capivasertib + paclitaxel arm.

In 2019, AKT1 and also PTEN mutations in BC were rated with the ESCAT scale tier IIB [208], because clinical trial(s) showed objective responses in patients presenting the alteration, but without conclusive data on outcome. Consequently, AKT1 mutations are not included in ASCO, ESMO or AGO recommendations at present.

### 8.7. Immune Checkpoint Inhibitors (ICI)

In recent years, immune checkpoint inhibitors (ICI), like the PD-1i or PDL-1i, were under extensive evaluation in a variety of solid tumors including BC. Multiple biomarkers were discussed to have predictive value for ICI treatment, reviewed with a special regard on blood-based biomarkers in 2020 [305]. The biomarker repertoire includes microsatellite instability (MSI), tumor mutational burden (TMB), circulating immune cells, HLAs, PD(L)-1 alterations and expression in/on circulating cells, EVs or via circulating nucleic acids and proteins, specific cfDNA mutations and T cell receptor (TCR) diversity [305]. In clinical practice, MSI and mismatch repair deficiency (MMR-D) profiling was recommended by the ESMO in 2020 for therapy selection of anti-PD-1 treatment [256] and MSI, MMR or TMB profiling was recommended by the ASCO in 2022 to determine the eligibility for dostarlimab-gxly (PD-1i) or pembrolizumab (PDL-1i) [271]. In Germany, only the PD(L)-1 expression profiling on the tissue by different scores (IC or CPS) is recommended to determine the eligibility for PD(L)-1i [98,148].

### 8.8. Tyrosine Receptor Kinase (TRK) Inhibition

In solid tumors, fusion genes of NTRK1/2/3 with other genes were reported with a prevalence of 0.3–0.5% [306]. In BC, NTRK fusions only appear in the very rare secretory BC [306]. The corresponding transmembrane proteins with kinase activity (namely Trk) are constitutively activated conferring oncogenic potential [208]. On the basis of two publications, which showed the effectiveness of Trk inhibitors in patients with different tumor entities including BC and NTRK fusions [307,308], Trk inhibitors for BC patients with NTRK fusions are recommended by the ESMO [256], the ASCO [271] and the AGO [148]. NTRK fusions are rated as ESCAT scale tier IC [208] (Table 12). At the end of 2022, the FDA approved blood-based evaluation of cfDNA using the FoundationOne (Cambridge, MA, USA) Liquid CDx test for the detection of NTRK1/2/3 fusion and subsequent Entrectinib therapy (Figure 2).

### 8.9. Androgen Receptor Inhibition

In imitation of prostate cancer treatment, it was discussed whether anti-androgen receptor treatment might be an option for TNBC patients. It was shown that AR protein expression is detectable in BC tumor tissue, however with a higher prevalence in ER-positive tumors (75–85%) than TNBC tumors (30%) [309,310,311]. Still, anti-AR therapy might be suitable in 30% of TNBC patients and detailed tumor tissue RNA expression analysis revealed some TNBC patients (16%) to be classified luminal androgen receptor (LAR) type [312,313]. Interestingly, the AR protein expression analysis on CTCs in the blood might be usable as predictive marker for anti-AR therapy selection in metastatic TNBC patients [314] and corresponding to the influence of the splice variant 7 of the AR transcript, CTC mRNA analysis showed a minority of early TNBC patients to potentially benefit from anti-AR therapy [315] (Table 13). Until now, anti-AR therapy is not recommended for treatment of BC patients.

### 8.10. Cylin Dependent Kinase 4/6 (CDK4/6) Inhibition

Although CDK4/6i revolutionized the treatment of HR-positive/HER2-negative MBCs, not all of these patients benefit (defined as PFS ≤ 6 months). Thus, the main goal is the search for a predictive marker to identify patients with de novo resistance. In this regard, the enumeration of CTCs at baseline using CellSearch did not show any significant association with PFS or clinical benefit of CDK4/6i [316]. A surrogate for the ctDNA fraction within the entirety of cfDNA called z-score was shown not to predict a risk of progression under CDK4/6i in case evaluated in the plasma samples at baseline [317]. A more recently published study, using a different method, showed a correlation of tumor fraction in cfDNA with PFS but not OS, in case evaluated before CDK4/6i initiation [84] (Table 14).

ESR1 mutations in cfDNA were candidates to predict worse PFS under CDK4/6i plus endocrine therapy. However, this hypothesis could not be proven, neither in studies with plasma samples collected outside of clinical trials [318] nor in studies with plasma samples from patients included in the MONARCH-2 [319] or PALOMA-3 trial [290,320,321,322]. Similarly, the potential of plasma PIK3CA mutations before CDK4/6i as predictive markers (shown for 30 patients [323] and in separate samples from the MONALEESA-7 trial [324]), could not be validated until now, although a large number of plasma samples from the MONARCH-2 and PALOMA-3 trials were studied [290,319,320].

In addition to PIK3CA and ESR1 alterations, CCND1 alterations were discussed to have predictive value for CDK4/6i which was not confirmed in the PALOMA-1 trial [325] nor in the MONALEESA-7 trials [324]. However, in the latter one, patients treated with palbociclib and fulvestrant with baseline KRAS mutations had a worse median PFS compared to patients with KRAS wild-type [324].

Although alterations in FGFR signaling pathway detected by tumor tissue analysis were shown to correlate with CDK4/6i resistance [298] and FGFR1 copy number alterations were detected in cfDNA of BC patients [326], the predictive value of FGFR copy number alterations in cfDNA for CDK4/6i has not been proven until now. In the MONALEESA-2 trial, consistent PFS benefit was shown independent of PIK3CA, TP53, ZNF703, FGFR1 and ESR1 mutations in the plasma or of CDKN2A, CCND1 and ESR1 mRNA levels or of Rb, Ki67 and p16 protein expression levels in tumor tissue, biopsied before therapy initiation [327]. Furthermore, in the MONALEESA-3 trial, ribociclib plus fulvestrant caused an outcome benefit compared to fulvestrant alone independent of baseline cfDNA status regarding PIK3CA, ESR1, TP53, CDH1, FGFR1, ZNF703 and WHSC1L1 mutations [328,329].

At the moment, the only promising predictive biomarkers for de novo resistance under CDK4/6i are RB1 alterations on the DNA and protein level in tissue and/or plasma. While patients with RB1 mutated tumors (prevalence 1.7%) did not have a significantly different median PFS under ribociclib treatment compared to placebo in the MONALEESA 2, 3 and 7 trials [199], in the PALOMA-3 trial, patients with RB loss (17.3% prevalence) at baseline had a significantly worse PFS under palbociclib plus fulvestant compared to RB wild-type patients [322]. In a small cohort of MBC patients treated with palbociclib (within the TREnd trial), gene expression regarding RB1 in single CTCs revealed a prolonged PFS, but not significantly prolonged [316]. Recently, a large study published the analysis of cfDNA via shallow WGS and DNA, RNA and protein analysis in tissue of patients treated with and without CDK4/6i [84]. The single evaluation of the RB locus on the 13q14.2 segment in the cfDNA did not show predictive value, however, the evaluation of a RB-LOH signature, consisting of 224 copy number features in the entire cfDNA genome showed a strong correlation with poor response and poor survival following CDK4/6i plus endocrine therapy [84]. The amplification of 2p (e.g., ETV6), 3q (e.g., PIK3CA), 8q (e.g., MYC), 20q (e.g., AURKA) and 21q (e.g., TMPRSS2 and ERG), and deletion of 2q (e.g., PARD3B), 4q, 5q, 12q, 13q (e.g., RB1), 15q and 17p are some of the 224 features included in the RB-LOH signature. The same signature evaluated in the tumor tissue was not significantly associated with PFS [84]. Importantly, the RB-LOH signature in the plasma did not only have predictive value for CDK4/6i but also prognostic value with regard to OS in MBC cohorts independent of CDK4/6i [84]. High mRNA expression levels of CDK4 in eVs, analyzed in baseline plasma samples from 40 HR-positive/HER2-negative advanced BC patients receiving palbociclib plus endocrine therapy correlated significantly with a longer PFS [330]. Thus, low CDK4 mRNA expression in EVs might be considered a de novo resistance marker. However, the ESMO guideline published in 2020 only recommended HR protein expression status in the tissue to identify patients who will benefit from CDK4/6i [256]. Disentangling the de novo resistance to CDK4/6i versus the de novo resistance to endocrine therapy, however, is still to be solved.

### 8.11. Predictive Biomarkers for BC Therapy Guidance

Table 15 lists the genomic alterations of strong clinical significance predictive for response to FDA-approved drugs in breast cancer. The level of evidence (LOE) and recommendations vary according to the consulting associations and are dynamic over time.

The 2022 ASCO guidelines do not recommend ctDNA and CTC analysis [271,331]. The ESMO 2021 guidelines stated that genomic profiling (independent of tissue or blood tests) should only be carried out in cases where the result will change treatment approaches or in case a patient will then be able to participate in appropriate clinical trials [6]. In 2022, the ESMO Precision Medicine Working Group mentioned tissue-based testing to remain the first choice, due to limitations of ctDNA assays in detecting fusion events and copy number changes [332]. However, ctDNA assays that are validated and are adequately sensitive can be used in routine clinical practice, but only if the constraints of the assays are considered [332]. These ctDNA assays are preferred when obtaining more rapid results is clinically essential or when tissue biopsies are either not suitable or not possible [332].

Although the German S1 guideline for tumor genetics from December 2021 published that LB can be used complementary to tissue-based approaches to identify actionable variants, they strengthened that tissue-based analysis is still superior to LB. The AGO recommended CTC quantification for prognosis and early therapy monitoring of MBCs but not for therapy guidance. Furthermore, CTC phenotype and cfDNA analysis is also not recommended by the AGO for these patients [148].

The FDA-approved tests listed in Figure 2 are evaluating predictive blood-based biomarkers in BC. BRCA1/2 germline mutation testing were the first to be approved from whole blood. The therascreen PIK3CA RGQ PCR was the first FDA-approved blood-based test to analyze somatic variants in BC and the FoundationOne Liquid CDx was approved to detect PIK3CA mutations as well. Two years later, the same test was also approved for the detection of NTRK fusions. Finally, the FDA approved the Guardant360 CDx to test for ESR1 variants in MBC patients.

In June 2023, the FDA started a pilot program to elaborate the possibilities of using laboratory developed tests for the identification of predictive biomarkers instead of only using FDA approved tests.

## 9. Liquid Biopsies for Therapy Monitoring in Breast Cancer

In the following, monitoring value is defined as (1) the association of a laboratory result from a blood sample drawn under therapy with the clinically and/or radiographically proven therapy response or (2) the association of a laboratory result from a blood sample drawn under therapy with the prognosis of the disease/therapy in the course of time.

A systematic review analyzing primary articles investigating the value of cfDNA in MBC to monitor response [333] concluded that—despite heterogeneous (pre-)processing procedures, techniques and designs—most studies found an association between ctDNA and treatment response. Another systematic review elucidating the monitoring value of CTCs and cfDNA identified more cfDNA studies that had albocicld as the inclusion criteria (52.7% of all primary articles) compared to CTC studies (17.6%) [149]. In total, 12.6% of all primary articles in this review evaluated the monitoring value of both, cfDNA and CTCs [149]. The latter review visualizes the diversity of analytes that might represent biomarkers for response to systemic treatment (Table 16).

### 9.1. Circulating Proteins

The circulating proteins CEA, CA 15-3, and CA 27-29 were recommended for therapy monitoring in 2015 by the ASCO [334]. However, their long half-lives might be one of the reasons for their low sensitivity for therapy response monitoring [158].

### 9.2. CTCs

CTC quantification was frequently shown to have monitoring value in the MBC setting, independent of the given therapy. It was shown that the CTC count itself by CellSearch evaluated 3–5 or 6–8 weeks after initiation of therapy was significantly associated with PFS and OS [168]. A decrease in CTC counts from baseline to a time point under therapy was related to an increased PFS and OS [335] and persistently high CTC counts from baseline to under therapy, despite radiologically proven therapy response, associated with worse outcome [336]. The DETECT V/CHEVENDO (NCT02344472) is currently recruiting HR-positive/HER-positive MBC patients who receive pertuzumab and trastuzumab, either in combination with chemotherapy or endocrine therapy, to evaluate CTC quantification before and during the therapy to examine the prognostic and monitoring value of this blood-based evaluation.

Based on these results, in the SWOG S0500 trial, MBC patients with persistently increased CTC counts after 21 days of therapy were randomly assigned to continue initial therapy or change to an alternative chemotherapy [337]. The data evaluation showed no significant benefit from the early switch to an alternative chemotherapy in MBC patients with persistently high CTC counts after 21 days under initial therapy [337]. In the CirCe01 trial [338], MBCs were randomized either in the CTC-driven or the standard arm. In the CTC-driven arm, CTC counts via CellSearch were assessed at baseline and after the first cycle of therapy. Response to therapy was defined by CTC counts as ≥70% decrease in CTC number from baseline to completion of the first therapy cycle or an absolute number of ≥5 CTC per 7.5 mL blood after the first therapy cycle [339]. Patients not showing these CTC-driven response criteria were exposed to an early therapy switch. Data evaluation of the CirCe01 trial, however, revealed no significant prolonged OS in the CTC-driven arm compared to the arm with standard therapy response evaluation [338].

Molecular characterization of CTCs was found to have monitoring value. Comparison of the number of apoptotic CTCs from baseline to under therapy revealed a 50% apoptotic CTC reduction to differentiate between patients showing stable versus progressive disease [340] and in case the apoptotic CTC number decreased from baseline to under therapy by less than 10%, progressive disease was identified with 74% specificity [340]. A significant decrease in HER2-positive CTCs was only detected in MBC patients responding to anti-HER2 treatment with lapatinib, but not in patients progressing under lapatinib [341]. In case of anti-RANKL therapy with Denosumab in MBC patients, the increase in RANK-positive CTCs from baseline to day 2 [342] as well as the persistence of RANK-positive CTCs was related to a longer time to progress of the bone metastasis [342]. The persistence of CTCs overexpressing EpCAM, MUC1 or HER2 transcripts under therapy in MBC patients correlated with shorter OS [174]. CTC overexpression signals were related to the staging result at the time of blood draw in MBC patients and revealed 74% of all patients with progressive disease to have CTCs overexpressing either EMT markers or the stem cell marker ALDH1 in contrast to only 10% of patients with stable disease [173]. Similarly, it was shown that the overexpression of ERBB2, ERBB3, and ERCC1 alone or in combination with AURKA in CTCs of MBCs was significantly more prevalent in patients showing progressive disease at the time of blood draw compared to patients with stable disease [343]. Identification of CTCs with overexpression of ERBB2, ERBB3, and ERCC1 alone or in combination with AURKA during therapy in MBCs was furthermore related to a shorter OS [343]. In more detail, ERBB2 overexpression in CTCs was only detected in patients not treated with anti-HER2 therapy and was related to therapy failure at the time of blood draw and to a reduced OS [343]. The same group further showed similar results using a different CTC gene expression panel [344]: patients with progressive disease at the time of blood draw were more likely to have CTC overexpression signals than patients with stable disease. Interestingly, two different gene expression pattern in CTCs were shown for patients with progressive disease (with high prevalence of *ESR*, *MUC1*, *AURKA*, *RAD51*, *TOP2A*, *ADAM17*, *SCGB2A2*, *KRT19*, and *EPCAM* overexpression), but a homogeneous expression pattern in patients with stable disease [344]. ERBB2 and/or ERBB3 overexpression in CTCs was significantly correlated with progressive disease at the time of blood draw [345].

In 2022, the ASCO stated that there are insufficient data to recommend CTCs to monitor response in MBCs [271]. In Germany, the AGO recommended CTC quantification to evaluate the early therapy response after three weeks in MBCs, but did not recommend the CTC quantification for therapy switch [148].

### 9.3. CTCs and EVs

Besides CTC overexpression signals, EV overexpression signals were studied. A stronger correlation of ERBB2 and ERBB3 signals in CTCs and EVs with disease progression was identified compared to ERBB2 and ERBB3 signals in CTCs alone, revealing a synergistic value of CTCs and EVs for therapy monitoring [345]. Interestingly, mTOR overexpression signals in EVs of MBCs under therapy were related to consecutive therapy failure [345] while mTOR overexpression in CTCs was related to patients showing therapy response over at least six months.

### 9.4. cfDNA

In the TBCRC 005 study, a 9-marker cfDNA methylation assay was shown to forecast disease progression three months earlier than radiographic staging in MBC patients [346].

A more than 50% reduction in genomic instability number (GIN) from low-pass WGS of cfDNA at baseline to one week under therapy was shown to associate with the stable disease proven by staging after 3 months and also with OS in a cohort of 25 MBC patients [157]. A rise in GIN from baseline to two weeks under therapy was associated with poor response, evaluated three months after therapy initiation by staging [157]. Another approach to identifying gains or losses of chromosomal material in the cfDNA (the mFAST-SeqS), output defined as z-score, showed that the comparison of z-scores at baseline and under therapy (z-score trajectories) has monitoring value in HR-positive/HER2-negative MBC patients treated with CDK4/6i [317].

The LOTUS and INSPIRE trials documented that the mean allele frequency dynamics from baseline to a time point under therapy related to therapy response at the time of blood draw or to PFS and OS in MBCs treated with different therapy regimens [347,348,349,350,351,352]. In the POSEIDON and SUMMIT trials, early evaluation of ctDNA changes forecasted the radiologic treatment response and the emergence of specific mutations correlated with clinical drug resistance [251,353]. More specifically, allele frequency of HER2 mutations in cfDNA decreased under pan-HER inhibitor neratinib in the SUMMIT trial, but increased upon radiographically proven progression [251]. In HER2-positive MBCs with brain metastases, the dynamic changes in ctDNA in CSF and plasma under therapy revealed decreased allele frequencies in the plasma to be consistent with extra-CNS disease control and increased allele frequencies in the CSF to be related to poor treatment benefit in CNS [354]. In the INSPIRE trial, TNBC patients, among patients with other tumor entities, were treated with pembrolizumab and ctDNA level changes from baseline to six weeks under treatment forecasted the therapy benefit [355]. In all patients who responded to therapy, ctDNA clearance was detected before the visible radiological response [355].

The PALOMA-3 trial demonstrated that the cfDNA level decrease, in general, was not valuable to forecast PFS or OS. cfDNA ESR1 mutations were also a weak marker for monitoring whereas cfDNA PIK3CA mutation dynamics had significant monitoring value [259]. A decrease in PIK3CA mutations in the cfDNA of palbociclib-treated patients from baseline to two weeks correlated significantly with increased PFS and long-term clinical benefit [259]. It was questioned whether a persistently high PIK3CA mutation level in cfDNA after two weeks of CDK4/6i might indicate a PI3Ki to be more effective [259]. However, a corresponding intervention trial has not been initiated. In the ALCINA trial, cfDNA evaluation at day 15 under palboclib plus fulvestrant showed a decrease in all patients independent of their PFS [356]. However, on day 30, undetectable cfDNA mutations (PIK3CA, TP53 and AKT1 studied) were associated with improved PFS [356]. In another evaluation of the ALCINA trial, the decline in cfDNA ESR1 mutations from baseline to day 15 under palbociclib plus fulvestrant was validated [357] and the informative value regarding PFS forecast of cfDNA mutation analysis at day 30, in comparison to baseline, was shown for ESR1 mutations [357]. ESR1 mutation detection in the plasma under first-line AI treatment revealed a direct association with progressive disease with 100% specificity [358]. ESR1 mutations were detectable prior to progression with a median lead time of 110 days [358].

In the interventional PADA-1 trial, cfDNA ESR1 mutations were questioned as both predictive and monitoring markers. Rising allele frequencies of cfDNA ESR1 mutations were used to identify patients with no radiographically proven progressive disease under palbociclib and AI suitable for therapy switch to fulvestrant plus palbociclib [276]. Data evaluation demonstrated a significant clinical benefit with regard to PFS in case the therapy switch was conducted from AI plus palbociclib to fulvestrant plus palbociclib in patients with rising ESR1 mutations detectable under therapy [276]. In accordance with the PADA-1 trial, the SERENA-6 trial (NCT04964934) is currently recruiting patients to receive letrozole and CDK4/6i, who will be confronted with a switch to the SERD AZD9833 plus CDK4/6i in case no progression is visible by radiographic staging but rising ESR1 mutations in the plasma are detectable.

Before the publication of the PADA-1 trial results, the ESMO did not recommend ctDNA analysis in general for monitoring purposes [256] and also rejected ESR1 mutation detection for monitoring or switch from AI to fulvestrant [256]. In the same year as the publication of the PADA-1 trial, ASCO and ESMO did not recommend monitoring MBC therapy responses by (ESR1) cfDNA detection [271,332]. The ESMO reported the need for validating whether cfDNA dynamics have clinical utility, the need to show improved OS, the need to define optimal time points for blood draw and optimal thresholds [332].

### 9.5. CTC and cfDNA Results and a Multimodal Approach

Monitoring on the basis of ctDNA evaluation was shown to have a higher sensitivity and higher correlation with tumor burden compared to CA 15-3 and CTC evaluations in MBCs [359]. A multimodal approach evaluating CTC mRNA, EV mRNA and cfDNA mutations emphasized the additive value of these analytes in MBC treatment monitoring, noting unique features of each analyte for disease surveillance [360]. In more detail, it was shown that the presence of either ERBB3 overexpression signals or ERBB2 overexpression signals in CTCs were related significantly to the staging result at the time of blood draw [360]. Additionally, the combined evaluation of ERBB3 in all three analytes associated with therapy response at the time of blood draw [360]. Dynamics from one time point to the next time point were more informative than single time point evaluations. In this regard, the overexpression signals in EVs were the most dynamic ones during therapy and newly occurring ERCC1 overexpression signals in EVs from one time point to the next had a specificity of 97% but sensitivity of 18% to determine therapy response [360]. The accuracy for detecting disease progression was 70% and 66% for PIK3CA and ESR1 variant appearances and the combined evaluation of ESR1 or PIK3CA allele frequency development was significantly correlated with disease progression [360]. Analysis of index patients indicated that the multimodal approach might cover the range of inter-patient heterogeneity. The three blood analytes complement each other, as specific EV signals were shown to be the most dynamic markers, the most accurate monitoring markers originated from the CTC fraction and the actionability of detected cfDNA variants might enrich the monitoring value by their predictive value relevant for therapy switch to a specific targeted therapy in the next line [360].

## 10. Challenges for Liquid Biopsy in Breast Cancer Management

The translation of liquid biopsies into clinical practice is still challenging. The physiological processes underlying the development and release of the biomarkers into the blood stream is mostly unknown as well as the lack of knowledge of the entirety of their physiological characteristics. CTCs often differ from tumor cells in the tissue and thus, CTCs might not reflect the actual intratumoral heterogeneity. It is a challenge for the isolation process that CTCs have different sizes, which might in some cases be similar to leukocytes. The rareness of CTCs is another challenge in the isolation process and therapeutic guidance based on CTC counts is limited to the few patients with high CTC counts. Processes and kinetics of cfDNA shedding/releasing and degradation are not fully understood. It was speculated that not all BC metastases release ctDNA into the blood stream in measurable quantities [361], again stating that the circulating biomarkers might not present the tumoral heterogeneity in its entirety. In case the entirety of cfDNA originates from dying cells, it is still an open question why cfDNA carries information about resistant tumor cells. Further challenges of cfDNA are their highly fragmented nature as well as the background of cfDNA from non-malignant cells including cfDNA harboring alterations due to clonal hematopoiesis (CHIP) [362]. EV research intensified to clarify the diverse mechanisms of selection and packaging cargo into the Evs, however, many questions still remain. More sensitive methods for purification without contaminants should be developed and quality standards should be adhered to [363].

The nature of blood-based testing involves important pre-analytical issues to be standardized including patients’ characteristics and lifestyle, timing of the blood draw, sample collection tubes, sample storage, sample processing and analyte isolation methods [364].

The European Committee for Standardization (CEN) and their specific Technical Committee 140 for in vitro diagnostic medical devices (CEN/TC 140) have already published standard operating procedures for the isolation of cfDNA from plasma (CEN/TS 16835-3:2015 Part 3). The American Society of Clinical Oncology and Colleagues of American Pathologists also published a statement including protocols regarding pre-analytical issues for cfDNA analysis [365]. While cfDNA isolation methods were compared in detail [366], the isolation methods for CTCs and EVs are still under debate in the field. In general, the current CTC isolation methods mostly enrich CTCs with a high background of leukocytes, which makes the data interpretation difficult.

Sensitivity and specificity as well as the limit of detection have to be improved. The limit of detection is especially a challenge in the field of MRD detection. The methods should be accurate, robust, reproducible and cost-effective. The establishment of clinically relevant thresholds to stratify patients for different treatments is needed [193]. Handling of negative results must be thought through [367]. In the context of NGS, bioinformatical pipelines, annotation of variants and interpretation of variants have to be standardized.

Another challenge is the design and conduction of well-designed, multicenter, randomized, large-scale, biomarker-stratified trials, with robust statistical methods, to prove the clinical utility of liquid biopsies. At the moment, only a few interventional trials based on LB stratification have been conducted and most failed to prove clinical utility, meaning that patients benefit due to biomarker usage [368,369]. Besides the challenge of a sophisticated study design, the choice of appropriate endpoints is essential.

Without proven clinical utility, reimbursement is not achievable, which is one of the major obstacles hampering translation into standard care [370], although cost-effectiveness studies showed a positive effect of LB testing. Recently, a rise in the number of reimbursed LB tests by private and public payers was observed [371], probably in linear association with the number of studies showing the clinical utility of blood-based testing.

To push the translation of liquid biopsies into clinical practice, consortia of researchers from academia, industry, regulatory agencies and the public, both in the United States (BloodPAC) and Europe (Cancer-ID and ELBS), were put in place.

## 11. Conclusions and Future Directions

To summarize this comprehensive review article, body fluids in the BC setting, mostly blood, include a diversity of analytes relevant in therapy management. Tremendous progress in the development of sensitive technologies has enabled the detection and analysis of tumor material in body fluids. Fields of application and consequences of LB testing in BC are early BC detection, detailed BC diagnostics, therapy (de-) escalation and/or therapy switch in the early BC setting, minimal residual disease detection, therapy guidance, prognostication and therapy monitoring. Currently, six FDA-approved tests exist for therapy guidance using predictive biomarkers from the blood (Figure 2). The number of genomic alterations recommended for therapy guidance in BC has steadily increased, with PIK3CA mutations, ERBB2 amplifications, NTRK fusions, MSI, TMB and ESR1 variants currently available (Table 15). Besides therapy guidance and prognostication, liquid biopsies have huge potential but are not recommended for further use in the BC setting by the ASCO, ESMO and AGO.

## Figures and Tables

**Table 1:** Liquid Biopsies for early detection.

Early Detection			
Specific Analyte	Clinical Setting	Conclusion	Reference
**(1) cfDNA**			
targeted cfDNA mutation analysis combined with the evaluation of circulating proteins	DETECT-A study, *n* = 10,006	specificity of 99% and a sensitivity of 33% to detect eight tumor types, including BC	10.1126/science.aar3247
different dimensions for cfDNA analysis	CCGA, NCT02889978	Whole genome bisulphite sequencing for methylation analysis and targeted sequencing single nucleotide variants with paired white blood cell background removal showed the lowest limit of detection	10.1016/j.ccell.2022.10.022
targeted methylation cfDNA sequencing	CCGA sub-study 2 and the STRIVE study (NCT03085888)	The specificity within all covered cancer entities was 99.3% and the sensitivity to detect BCs with stage I disease was <10% (stage I), 50% (stage II) and >80% (stage III or IV).	10.1016/j.annonc.2020.02.011
targeted methylation cfDNA sequencing panel of >100,000 regions	CCGA sub-study 3	specificity was 99.5% (low false-positive rate of 0.5%). Overall sensitivity across cancer classes and stages was 51.5%, but for BC only 30.5% across all stages	10.1016/j.annonc.2021.05.806
targeted methylation cfDNA sequencing panel of >100,000 regions	CCGA sub-study 3 in patients with symptoms only	increased overall sensitivity of 64.3% (52.8% for BC) and the overall accuracy of the cancer site of origin prediction in true positives was 90.3%	10.1200/PO.22.00679
targeted cfDNA methylation-based MCED test, here referred to as Galleri (MCED-Scr; 30,000 CpG fragments covered)	PATHFINDER study (NCT04241796), in adults with elevated cancer risk	Adding Galleri test to standard of care screening more than doubled the number of cancers detected. Half of the cancers detected with the blood test, were stage I or II. Accuracy of tissue of origin was 97.1%. In total, 71% of participants with a Galleri detected cancer had cancer types with no routine screening test available. PPV was 43.1% and the false-positive rate was less than 1%	10.3390/diagnostics12051244 and ESMO Congress 2022
cfDNA concentration	61 patients with breast cancer, 33 control patients with benign breast diseases and 27 healthy control individuals	cfDNA concentration in the BC patients was significantly higher than that in the control patients or healthy control.	10.1016/j.canlet.2005.11.027
cfDNA *PIK3CA* mutations		sensitivity of 93.3% and a specificity of 100% for detecting early-stage BC	10.1158/1078-0432.CCR-13-2933
cfDNA methylation (pyroseq of 3 genes: SPAG6, PER1 and ITIH5; SNiPER)	plasma cohort (*n* = 125)	64% sensitivity for breast cancer detection using SPAG6, PER1 and ITIH5	10.18632/oncotarget.27303
cfDNA methylation (one gene: APC)	meta-analysis of 12 studies	low sensitivity (20%) but high specificity (96%) for detecting breast cancer	10.1111/1759-7714.12580
cfDNA methylation (2 genes)		94.1% sensitivity	10.1016/j.ygyno.2010.04.016
cfDNA methylation (8 genes)		90% sensitivity	10.1371/journal.pone.0016080
cfDNA methylation (3 genes)		greater sensitivity than the serum markers CEA and/or CA15-3	10.1007/s10549-011-1575-2
cfDNA methylation (EGFR and PPM1E promoter)		patients with BC had significantly higher methylation levels than healthy controls	10.1007/s13277-016-5190-z
cfDNA integrity index		patients with confirmed malignancy had significantly greater DNA damage than those with benign breast lesions and healthy controls	10.1007/s13277-015-4624-3
cfDNA mutations in breast milk	10 women diagnosed with BC during pregnancy and 9 diagnosed during breastfeeding: 12 healthy donors	Variants in cfDNA from breast milk detected in 87% of the cases, while undetected in 92% of matched plasma. Overall clinical sensitivity of 71.4% and specificity of 100%. In two cases, ctDNA was detectable in BM collected 18 and 6 months prior to standard diagnosis.	10.1158/2159-8290.CD-22-1340
**(2) CTCs**			
CTCs by nuclease-activated probe technology		discrimination between BC patients and healthy controls	10.1016/j.omtn.2017.08.004
**(3) EVs**			
EVs with specific proteomic profiles, including immunoglobulins	426 human samples	95% sensitivity/90% specificity in detecting cancer	10.1016/j.cell.2020.07.009
EVs with CD82		quantification for early diagnosis of BC	10.1002/mc.22960
EVs including unique tRNAs and miR-10b and miR-21		EVs BC patients contain miR-10b and miR21 and unique tRNA (in contrast to healthy controls that do not convert pre-miRNA of these two types)	10.1158/1541-7786.MCR-14-0533
EVs including miR-21 and miR-1246		significant higher concentration in BC patients than in healthy controls	10.1186/s13058-016-0753-x
EVs with long non-coding RNA (lncRNA H19)		H19 expression in EVs was significantly upregulated in the serum of patients with BC as compared to patients without malignancy	10.2147/OTT.S243601
**(4) other blood analytes**			
circulating miRNAs (8 miRNAs)	Serum samples from 116 malignant breast lesions and 64 benign breast lesions	area under the curve (AUC) of 0.9542 with eight-miRNA signature	10.3390/cancers11121872
circulating miRNAs (5 miRNAs)		Combination of miR-1246, miR-1307-3p, miR-4634, miR-6861-5p, and miR-6875-5p was shown to detect early-stage BC with sensitivity of 97.3%, specificity of 82.9% and accuracy of 89.7%.	10.1111/cas.12880
circulating miRNAs	55 patients with metastatic breast cancer and 20 healthy donors	miR-21, miR-146a, and miR-210 could discriminate patients from healthy individuals	10.1373/clinchem.2015.253716
proteins (afamin, apolipoprotein E, alpha-2-macroglobulin and ceruloplasmin)	68 women diagnosed with BC within three years after enrollment, with 68 matched controls	Afamin, apolipoprotein E and ITIH4 were found in higher concentration in pre-diagnostic breast cancer (*p* < 0.05), while alpha-2-macroglobulin and ceruloplasmin were lower (*p* < 0.05).	10.1186/1471-2407-11-381
proteins (integrin subunit alpha, Filamin A, Ras-associated protein-1A and Talin-1)	20 patients with BC and 20 female control individuals with positive mammograms and benign pathology at biopsy	4 proteins classified breast cancer patients with 100% sensitivity and 85% specificity (AUC of 0.93)	10.1186/s13058-020-01373-9
proteins (Cyr 61) in plasma	544 patients BC and 427 healthy controls	specificity of 99.0% and sensitivity of 80.0% for cancer detection	10.1093/clinchem/hvab153
volatile organic compounds in the urine		sensitivity of 93.3% and specificity of 83.3%	10.1038/s41598-021-99396-5
**(5) multiple blood analytes**			
cfDNA mutation and cfDNA methylation as well as circulating miRNA information	205 patients with stage I, II, or III cancer prior to cancer therapy and 15 healthy controls	combination of three different analytes could improve the sensitivity for cancer detection	10.3390/cancers14020462
cfDNA fragmentation combined with cfDNA mutation analysis		sensitivity 91% and specificity 98% with combined workflow	10.1038/s41586-019-1272-6
cfDNA integrity, in combination with the detection of CTCs	84 patients with no-distant metastatic BC and 30 patients with benign breast tumors	Combination of CTCs with cfDI: false positive rate 10.71% andarea under the curve value 0.68.	10.4149/neo_2017_417
combination of circulating mRNAs and a protein		Eight mRNAs (S100A8, GRIK1, GRM1, H6PD, IGF2BP1, CSTA, MDM4,and TPT1) and the CA6 protein were able to distinguish BC patients and healthy controls. Diagnostic accuracy: 92% (sensitivity of 83% and specificity of 97%).	10.1371/journal.pone.0015573

**Table 2:** Liquid Biopsies for detailed BC diagnostic.

Diagnostic		
Specific Analyte	Conclusion	Reference
**(1) cfDNA**		
nucleosome position and accessibility of cfDNA	differentiate ER-positive from ER-negative MBCs	10.1038/s41467-022-35076-w
ctDNA fraction	High ctDNA fraction itself has already been shown to correlate with TNBC status, and also high tumor grade and metastatic status	10.1038/s41523-021-00319-4
**(2) CTCs**		
CTC number	A significantly increased number of CTCs, determined by CellSearch, before therapy was reported for MBCs with lobular compared to ductal histology	10.3390/cells9071718
AR on CTCs	androgen receptor (AR) expression on CTCs was correlated with bone metastasis	10.1158/1541-7786.MCR-17-0480
number of apoptotic CTCs	higher number of apoptotic CTCs was detected in early in contrast to metastatic BC patients	10.1158/1535-7163.MCT-12-1167
**(3) EVs**		
EV miRNA-373	EV miRNA-373 was increased in the blood of TNBCs patients compared to patients with other BC subtypes	10.18632/oncotarget.2520
EV mRNA	Profiling of PAM50 transcripts in EVs showed good concordance with tissue results.	10.1021/acs.analchem.3c00624
45 miRNAs in EVs	panel of 45 miRNAs detected in plasma EVs of BC patients differentiated HER2-positive from TNBC patients	10.1186/s12916-018-1163-y
EV miR-21	MBCs were shown to have significantly higher EV miR-21 levels compared to BC patients with no metastases	10.1186/s13058-019-1109-0
EV miR-223-3p	EV miR-223-3p was significantly increased in invasive ductal carcinoma patients compared to subjects with ductal carcinoma in situ (DCIS)	10.3892/ol.2018.8457
**(4) other blood analytes**		
Circular RNA, circ_0001785	circ_0001785 is proposed to be correlated with distant metastasis and histology	10.1016/j.cca.2017.10.011
**(5) multiple blood analytes**		
56-gene cfDNA Panel (including SNV, CNV, MSI, TMB analysis) and ER and HER2 protein expression and ERBB2 amp in/on CTCs	to subtype MBC	https://www.epicsciences.com/press-releases/epic-sciences-announces-medicare-coverage-for-breast-cancer-focused-ctdna-gene-panel/?goal=0_8c04e2abda-5ca6438936-71670485&mc_cid=5ca6438936

**Table 3:** Liquid Biopsies for therapy (de)escalation.

Therapy (De)Escalation			
Specific Analyte	Clinical Setting	Conclusion	Reference
**(1) cfDNA**			
ctDNA quantity	before neoadjuvant therapy	ctDNA quantity predicted the risk of relapse and OS	10.1016/j.ctrv.2022.102362
ctDNA quantity	before neoadjuvant chemotherapy in TNBC patients	predicting the risk for recurrence	10.18632/oncotarget.23520
PIK3CA and/or TP53 mutation detection in cfDNA	NeoALTTO trial (before neoadjuvant anti-HER2 treatment in HER2-positive BC patients)	PIK3CA and/or TP53 mutations in cfDNA correlated with lower pCR rates	10.1158/1078-0432.CCR-18-2521
cfDNA methylation of GASTP1, RASSF1A and RARB2	before neoadjuvant treatment in early BC patients	cfDNA methylation of GASTP1, RASSF1A and RARB2 was associated with OS independent of pCR	10.1159/000342083
ctDNA clearance	I-SPY 2 study (neoadjuvant therapy with an AKT inhibitor); 84 high risk early BC patients; from baseline to three weeks after therapy initiation	ctDNA clearance from baseline to three weeks was related to an increased pCR rate and even in patients with no pCR, the ctDNA clearance within the first three weeks of therapy was correlated with improved survival compared to patients achieving no pCR and no ctDNA clearance	10.1016/j.annonc.2020.11.007
mutations in cfDNA	under neoadjuvant therapy	Detection of mutations in cfDNA based on tumor-informed personalized assays under neoadjuvant therapy were correlated with a lower chance of pCR	10.1126/scitranslmed.aax7392
**(2) CTCs**			
CTC detection by CellSearch	NeoALTTO trial (before neoadjuvant anti-HER2 treatment in HER2-positive BC patients)	CTC detection resulted in numerically lower pCR rates (pCR in 27.3% patients with detectable CTCs and pCR in 42.5% with no detectable CTCs).	10.1016/j.breast.2013.08.014
CTC detection	GeparQuattro trial (before neoadjuvant chemotherapy)	CTC detection correlated significantly with disease-free (DSF) and OS	10.1158/1078-0432.CCR-17-0255
CTC quantity by CellSearch	BEVERLY-1 and -2 trials (before neoadjuvant chemotherapy)	CTC quantity did not show any correlation to pCR rates, but CTC detection was associated with significantly decreased DFS and OS	10.1093/annonc/mdw535
CTC detection	2000 early BC patients (before neoadjuvant therapy)	presence of CTCs was an independent predictor of poor DSF, distant disease free (DDSF) and OS.	10.1158/1078-0432.CCR-15-1603 and 10.1093/jnci/djy018
**(3) EVs**			
EV miRNA (miR-30b, miR-328 and miR-423)	before neoadjuvant therapy in BC patients	levels of specific EV miRNA (miR-30b, miR-328 and miR-423) forecast pCR	10.3390/curroncol29020055
miR-141, miR-34a, miR-182 and miR-183 in EVs	after the first dose of neoadjuvant therapy	miR-141, miR-34a, miR-182 and miR-183 in EVs after the first dose of neoadjuvant therapy predicted pCR/non-pCR	10.3390/curroncol29020055
**(4) other blood analytes**			
miRNAs in plasma	TNBC patients before neoadjuvant therapy	miRNAs in plasma correlate with relapse and OS	10.1158/1078-0432.CCR-14-2011
miRNAs in plasma	before neoadjuvant therapy	miRNAs in plasma correlate with pCR	10.3390/cancers12071820 and 10.1007/s10549-022-06642-z
miR-145 in plasma	HER2-positive BC, before neoadjuvant therapy	Reduced miR-145 levels were related to pCR in HER2-positive BC	10.1097/SLA.0000000000005613
let7a in plasma	luminal BC, before neoadjuvant therapy	let7a correlated with pCR	10.1097/SLA.0000000000005613
level of circulating nucleosomes	before neoadjuvant therapy in early BC patients	level of circulating nucleosomes had prognostic value	10.1016/j.canlet.2013.04.013
circulating miR-148a-3p and miR-374a-5p	from baseline to two weeks of trastuzumab-based neoadjuvant chemotherapy in the NeoALTTO trial	increased levels of circulating miR-148a-3p and miR-374a-5p from baseline to two weeks were related to pCR	10.3390/ijms21041386
thymidine kinase activity in the plasma	early under neoadjuvant treatment	prognostic value	10.1016/j.esmoop.2021.100076

**Table 4:** Liquid Biopsies to anticipate minimal residual disease.

Minimal Residual Disease Detection			
Specific Analyte	Clinical Setting	Conclusion	Reference
**(1) cfDNA**			
ctDNA dynamics	before and after neoadjuvant treatment	ctDNA clearance during neoadjuvant therapy was informative regarding the existence of MRD	10.1158/1078-0432.CCR-21-3231
ctDNA dynamics by personalized mutation assays	I-SPY 2 study (neoadjuvant therapy with an AKT inhibitor); 84 high risk early BC patients; from baseline to end of neoadjuvant treatment	cfDNA clearance from baseline to the end of treatment correlated with a pCR. Patients with ctDNA detection after therapy showed a significantly increased risk for metastatic recurrence. In particular, patients not achieving a pCR but with no ctDNA detection after therapy had an excellent outcome, similar to the patients who achieved a pCR.	10.1016/j.annonc.2020.11.007
ctDNA dynamics by targeted digital sequencing	before and after neoadjuvant treatment	patients with a pCR showed a larger decrease in ctDNA during neoadjuvant therapy compared to the patients with no pCR	10.1126/scitranslmed.aax7392
ctDNA dynamics by BC-specific methylation pattern	before and after neoadjuvant treatment	ctDNA persistence even after neoadjuvant therapy indicated the existence of MRD	10.1016/j.annonc.2019.11.014
cfDNA integrity	before and after neoadjuvant treatment	longitudinal cfDNA integrity analysis indicated tumor shrinkage	10.4149/neo_2017_417
cfDNA integrity index	after neoadjuvant treatment	Patients who achieved a pCR, but showed an reduced cfDNA integrity index after neoadjuvant therapy had a higher risk for distant metastases	10.4149/neo_2017_417
ctDNA detection by mutation analysis	after neoadjuvant treatment	prognostic value of ctDNA detection by mutation analysis in all BC subgroups after neo-adjuvant therapy	10.1016/j.ctrv.2022.102362 and 10.1001/jamaoncol.2019.1838 and 10.1038/s41523-017-0028-4 and 10.15252/emmm.201404913 and 10.1126/scitranslmed.aab0021 and 10.1001/jamaoncol.2020.2295
ctDNA concentration and presence	after neoadjuvant treatment	ctDNA presence after neoadjuvant therapy was detected in 12/13 patients with no pCR, but also in 5/9 patients achieving a pCR. ctDNA concentration but not ctDNA presence after neoadjuvant therapy was significantly correlated with a pCR	10.1126/scitranslmed.aax7392
ctDNA detection	IMPASSION031 trial, TNBC patients, after neoadjuvant treatment	Patients achieving a pCR and who had no detectable ctDNA showed the best DSF and OS while the non-pCR cohort could be differentiated by ctDNA presence in patients with increased DSF and OS (ctDNA negative) and patients with worse DSF and OS (ctDNA positive)	10.1016/esmoop/esmoop101220
ctDNA detection by personalized mutation sequencing panels	in the follow-up	sensitivity of 89% for MRD detection with a lead time of up to 24 months (median 8.9 months) with a specificity of 100% with none of the non-relapsing patients being ctDNA-positive	10.1158/1078-0432.CCR-18-3663
ctDNA detection by patient-specific digital droplet PCR (ddPCR) panels	within one year after surgery	MRD was detected with 19% sensitivity and median lead time from first positive test to recurrence was 18.9 months	10.1158/1078-0432.CCR-19-3005
ctDNA detection by RaDaR assays	high-risk HR-positive, HER2-negative BC patients with no evidence of recurrence five years after diagnosis, serial blood analysis	RaDaR assays identified all patients with distant metastatic recurrences (7.2%) with a median ctDNA lead time of 12.4 months. However, 2/8 patients with ctDNA-positive results had not had clinical recurrence. In total, 1.2% of patients had no MRD but local recurrence.	10.1200/JCO.22.00908
**(2) CTCs**			
CTC detection	before and after neoadjuvant treatment	presence of persisting CTCs correlated with shorter DSF and OS	10.1093/jnci/dju066
CTC detection	before and after neoadjuvant treatment	presence of persisting CTCs correlated with an increased risk of relapse	10.1245/s10434-015-4600-6
CTC number	in TNBC patients after neoadjuvant chemotherapy	one or more CTCs present after neoadjuvant chemotherapy predicted relapse and survival in TNBC patients	10.1245/s10434-015-4600-6
TWIST transcripts in CTCs	early BC patients after surgery and before adjuvant therapy	prognostication of DSF in early BC patients after surgery and before adjuvant therapy	10.3390/cells8070652
CK19 mRNA positive CTCs	during the first five years of BC follow-up	persistent detection of CK19 mRNA positive CTCs during the first five years of BC follow-up increased the risk of late relapse	10.1186/bcr2897
**(3) EVs**			
EV miR-21	before and after neoadjuvant treatment	Persisting high levels of circulating miR-21 after neoadjuvant treatment were associated with poor prognosis	10.1186/s13058-019-1109-0
**(4) other blood analytes**			
circulating miR-21	before and after neoadjuvant treatment	Persisting high levels of circulating miR-21 after neoadjuvant treatment were associated with poor prognosis	10.1007/s10549-022-06642-z
circulating miRNAs	NeoALTTO trial, after completion of neoadjuvant therapy	miR-185-5p, miR-146a-5p and miR-22-3p are prognostic marker independent of pCR	10.3389/fonc.2022.1028825
lymphocyte-to-monocyte ratio	after surgery and neoadjuvant therapy	lymphocyte-to-monocyte ratio was shown to be significantly associated with worse prognosis	10.2147/CMAR.S292048
expression of TLR4 on peripheral blood mononuclear cells	at the time point of surgery in early BC patients	the expression of TLR4 on peripheral blood mononuclear cells predicted high risk of relapse	10.3390/cancers14041053
**(5) multiple blood analytes**			
cfDNA and CTC analysis	TNBC patients after neoadjuvant treatment	MRD sensitivity was 79% with ctDNA analysis alone, 62% with CTC analysis alone and 90% with the combination of both analytes	10.1001/jamaoncol.2020.2295
CTC quantification, phenotypic, transcriptomic, and genomic profiling of CTCs as well as mutation and methylation profiling of cfDNA	early BC patients in the follow-up	Multimodal approach identified a relapse at least four years earlier than metastases could clinically be detected	10.1038/s41598-022-25400-1

**Table 5:** Liquid Biopsies for prognostication in the metastatic BC setting.

Prognostification in MBCs			
Specific Analyte	Clinical Setting	Conclusion	Reference
**(1) cfDNA**			
genome-wide cfDNA methylation	MBCs, at baseline or week four after therapy initiation and dynamics	Methylation pattern on genome-wide scale in cfDNA was shown to correlate with OS—even prognostic in case evaluated at week four after therapy initiation and dynamics from baseline to four weeks were informative about OS as well.	10.1200/JCO.2015.66.2080
cfDNA methylation (5 genes)	at baseline in MBCs	correlate with OS	10.1038/s41388-018-0660-y
copy number changes and genomic instability score from cfDNA	MBCs, at baseline, after one week and two weeks after treatment initiation	The genomic instability score at baseline, after one week and two weeks after treatment initiation were significantly associated with poor OS.	10.3390/cancers13061331
Tumor-derived cfDNA fractions	MBC	associated with clinical outcome (PFS and OS)	10.3390/cancers11081171
ctDNA abundance by mutation-specific ddPCR	BEECH trial, ER-positive advanced BCs, after four weeks of therapy	ctDNA abundance after four weeks of therapy revealed significant correlation with PFS	10.1093/annonc/mdz085
pathogenic or likely pathogenic variants in the cfDNA	MBCs	Higher number of pathogenic or likely pathogenic variants in the cfDNA associated with worse OS	10.1007/s00018-019-03189-z
mean variant allele frequency of cfDNA mutations	from baseline to cycle two in advanced BC patients treated with ICI	It was also described that a decrease in the mean variant allele frequency of cfDNA mutations in any of the 425 genes sequenced from baseline to cycle two was related to a longer PFS	10.1200/PO.22.00509
cfDNA ESR1 mutations	42 pre-treated MBCs	cfDNA ESR1 mutations were found to indicate worse OS and were associated with shorter duration of endocrine treatment effectiveness	10.18632/oncotarget.18479
cfDNA TP53 and/or PIK3CA	MBCs	TP53 and/or PIK3CA mutations detected in cfDNA of MBCs were shown to indicate worse OS	10.1016/S1470-2045(17)30376-5 and 10.1016/j.clbc.2016.05.004
cfDNA specific BRCA1 mutation	44 HR-positive/HER2-negative MBC	Specific BRCA1 mutation detected in the cfDNA was associated worse OS	10.1007/s00018-019-03189-z
**(2) CTCs**			
CTCs number by the CellSearch system	177 MBC patients before therapy initiation of any therapy in any therapy line	A cut-off of five CTCs in 7.5ml blood differentiated patients with good (mean 7.0 months) versus worse (mean 2.7 months) PFS and correlated significantly with OS	10.1056/NEJMoa040766
CTCs number by the CellSearch system	83 newly diagnosed, measurable MBC who were about to start their first line of systemic therapy	cut-off of five CTCs per 7.5ml blood was applied: 52% of patients had ≥ five CTCs and these patients had a decreased PFS and OS compared to the patients with no or less than five CTCs	10.1200/JCO.2005.08.140
CTCs number by the CellSearch system	meta-analysis including 1944 MBC pa-tients	significant association of CTC quantity (cut-off: 5 CTCs) regarding PFS and OS	10.1016/S1470-2045(14)70069-5.
CTC clusters	MBCs (first line treated)	presence of CTC clusters has additional prognostic value when compared to the single CTC quantification alone. number of CTCs within the clusters might also be related to OS	10.1007/s10549-016-4026-2 and 10.1186/s13058-018-0976-0
CTC detection by positive immunomagnetic selection and molecular characterization	MBCs	CTC presence was significant associated with PFS; patients with CTCs showing high PALB2 or MYC transcript expression had a shorter PFS and OS; patients with CTCs showing epithelial-stem cell like features also showed shorter PFS and OS	10.1007/s10549-008-0143-x and 10.3390/cancers11121941
CTC isolation by a microfluidic chip and molecular characterization	MBC patients before eribulin treatment	entirety of epithelial and mesenchymal CTCs, as well as only the epithelial or mesenchymal CTCs was related to OS	10.1007/s12032-019-1314-9
CTC isolation by density and EpCAM expression and molecular characterization	MBCs	univariate Cox regression model showed prognostic value for the presence of CTCs with either CK-19 overexpression, HER2 overexpression or CTCs with CD44high/CD24low or ALDH1high/CD24low features	10.3390/diagnostics11030513
HER2+ CTCs	HER2-negative MBC	reduced OS in case CTCs with strong HER2 staining were detectable	10.1016/j.esmoop.2021.100299
mitotic activity of CTCs	MBCs	characterization of CTCs with regard to their mitotic activity increased the hazard ratio for association with OS dramatically compared to CTC quantification itself	10.1186/s13058-016-0706-4
CTC mRNA profile	MBCs (first-line aromatase inhibitor (AI) treated patients vs. treated with other therapy regimens	a-8-gene predictor (EEF1A, PTRF, CXCL14, ERBB3, EGFR, PTPRK, KRT81, TWIST1) was published to be related to PFS in first-line aromatase inhibitor (AI) treated patients, while the same predictor was not related to PFS in MBCs treated with other therapy regimens	10.1186/s12885-016-2155-y
**(3) EVs**			
metastasis- and stemness-related mRNAs in EVs		A set of metastasis- and stemness-related mRNAs were higher expressed in EVs from BC patients with poor OS than in those patients with increased OS	10.18632/oncotarget.5818
**(4) other blood analytes**			
circulating miR-200 family members	at baseline of new line of systemic therapy in MBCs	miR-200a, miR-200b, miR-141, and miR-429 were shown to significantly correlate with progression-free survival (PFS)	10.1007/s00404-022-06442-2
thymidine kinase 1 (sTK1) in plasma	EFECT trial in MBCs, at baseline	prognostic value	10.1016/j.ejca.2019.04.002
LAMP2 protein levels in red blood cells	MBCs	related to OS	10.1016/j.mcpro.2022.100435
**(5) multiple blood analytes**			
CTC counts and total cfDNA level	MBCs	CTC counts and total cfDNA level were associated with OS in MBCs and thus, concluded CTCs and cfDNA to be equally valuable OS markers. the combined analysis of CTCs and cfDNA was more informative regarding OS than the sole analysis of one of the analytes	10.1158/1078-0432.CCR-16-0825 and 10.1186/s13058-019-1235-8 and 10.1016/j.ejca.2018.10.012
CTC counts by CellSearch and ctDNA identified by targeted NGS	UCBG COMET study (NCT01745757), first-line paclitaxel and bevacizumab	CTC counts and ctDNA had non-overlapping profiles and correlated in sole and also in combined analysis with OS	10.1038/s41523-021-00319-4
ESR1 variants in CTCs and cfDNA	MBCs	ESR1 variants in CTCs and cfDNA to indicate worse OS	10.3390/cancers12051084
SOX17 promotor methylation in cfDNA and CTCs	MBCs	SOX17 promotor methylation in cfDNA and CTCs to be of prognostic relevance	10.18632/oncotarget.18679
HER2+ CTCs and HER2+ EVs	MBCs	the heterogeneity of CTCs or EVs within one blood sample was shown to be inversely associated with OS	10.1186/s13058-020-01323-5
CTCs and disseminated tumor cells (DTCs) in the bone marrow	MBCs	DTCs as well as CTCs were significantly associated with worse OS, no significant association of DTC and CTC results	10.1007/s10549-014-3113-5
cfDNA, CTC genomic DNA, CTC mRNA and EV mRNA	ELIMA project, MBCs	additive value for prognostication: ‘ELIMA.score’ showed a significant correlation with OS with a decreased *p*-value when compared to each single analyte	10.1186/s13073-021-00902-1

**Table 6:** Liquid Biopsies for therapy guidance towards/against CTX.

Decision for/against CTX			
Specific Analyte	Clinical Setting	Conclusion	Reference
**(2) CTCs**			
CTC quantity by CellSearch	STIC CTC trial, first-line therapy selection in HER2-negative MBCs, before therapy initiation	Therapy selection was conducted either based on the CTC quantity or clinicians’ choice. In general, PFS and OS were equally distributed in all groups, however, in patients with no concordant stratification status (high risk by clinicians/low CTC number or low risk by clinicians/high CTC number), chemotherapy prolonged PFS and OS compared to endocrine therapy	10.1001/jamaoncol.2020.5660
**(3) EVs**			
Ubiquitin carboxyl-terminal hydrolase-L1 protein levels in EVs	before therapy initiation	Ubiquitin carboxyl-terminal hydrolase-L1 protein levels in EVs were shown to predict response to CTX	10.1002/jso.24614
**(4) other blood analytes**			
Circulating miR-125b	before therapy initiation	Circulating miR-125b was shown to predict response to CTX	10.1371/journal.pone.0034210

**Table 7:** Liquid Biopsies for therapy guidance towards/against PARP inhibition.

Decision for/against PARP Inhibition			
Specific Analyte	Clinical Setting	Conclusion	Reference
**(1) cfDNA**			
Somatic BRCA1/2 mutations (from cfDNA)	Olaparib Expanded trial, MBCs	The Olaparib Expanded trial also showed the effectiveness of Olaparib in MBC patients with somatic BRCA1/2 mutations. Olaparib therapy is rated as an option for MBC patients with somatic BRCA1/2 mutations (ESCAT scale IIA) by the ESMO guideline	10.1200/JCO.20.02151 and 10.1016/j.annonc.2021.09.019
**(4) other blood analytes**			
Germline BRCA1/2 mutations (from blood cells)	OLYMPIA trial, HER2-negative early BC patients	In early BC, gBRCA1/2 mutations are of prognostic value to achieve a pCR under chemotherapy and forecast DFS under PARP inhibition. Olaparib is recommended for early TNBC patients showing no pCR and harboring gBRCA1/2 mutations as well as for high risk gBRCA1/2 mutant HR-positive/HER2-negative early BC patients as proven in the OLYMPIA trial. Standard to test HER2-negative BC patients for gBRCA1/2 mutations (ESCAT scale IA	10.1159/000531578 and 10.1016/j.annonc.2022.09.159 and 10.1093/annonc/mdz036
Germline PALB2 mutations (from blood cells)	Olaparib Expanded trial, MBCs	The Olaparib Expanded trial also showed the effectiveness of Olaparib in MBC patients with germline PALB2 mutations. Olaparib therapy is rated as an option for MBC patients with germline PALB2 mutations (ESCAT scale IIA) by the ESMO guideline	10.1200/JCO.20.02151 and 10.1016/j.annonc.2021.09.019

**Table 8:** Liquid Biopsies for therapy guidance towards/against anti-HER2 therapy.

Decision for/against Anti-HER2 Therapy			
Specific Analyte	Clinical Setting	Conclusion	Reference
**(1) cfDNA**			
cfDNA ERBB2 mutations	MBCs	MBC patients with ERBB2 mutations were resistant to lapatinib, but sensitive to neratinib	10.1158/2159-8290.CD-12-0349 and 10.1038/nature25475 and 10.1158/1078-0432.CCR-17-0900
cfDNA ERBB2 mutations	plasmaMATCH trial, cohort B, MBC patients	Cohort B in the plasmaMATCH trial also showed a benefit of neratinib treatment in ERBB2 mutant MBC patients	10.1016/S1470-2045(20)30444-7
**(2) CTCs**			
CK19-positive CTCs	HER2-negative early BC patients	Treatment with trastuzumab prolonged the DFS in HER2-negative patients with CK19-positive CTCs present before and after adjuvant chemotherapy compared to observation. The fraction of patients with CK19-positive CTCs after trastuzumab treatment was reduced down to 14%, while observation led to 17.9% of patients with CK19-positive CTCs.	10.1093/annonc/mds020

**Table 9:** Liquid Biopsies for therapy guidance towards/against PIK3CA inhibition.

Decision for/against PIK3CA Inhibition			
Specific Analyte	Clinical Setting	Conclusion	Reference
**(1) cfDNA**			
cfDNA PIK3CA mutation	BELLE-2 trial, HR-positive/HER2-negative MBC patients progressing under aromatase inhibitor (AI) therapy	addition of buparlisib improved the PFS in PIK3CA mutant patients	10.1016/S1470-2045(17)30376-5 and 10.1016/j.ejca.2018.08.002
cfDNA PIK3CA mutation	SOLAR-1 study, MBCs	Worse PFS of PIK3CA mutant MBC patients was improved by the application of alpesilib to an extend of a PFS achieved in PIK3CA wildtype MBC patients, that did not benefit from alpesilib treatment.	10.1056/NEJMoa1813904
cfDNA PIK3CA mutation	HR-positive/HER2-negative MBC patients after progression under AI	Alpelisib in combination with fulvestrant for PIK3CA mutant HR-positive/HER2-negative MBC patients after progression under AI is recommended by the ESMO stating that ctDNA assessment for PIK3CA mutation analysis is an option besides mutational profiling in tissue samples. In patients with no available archival tumor tissue, ctDNA assessment is recommended. PIK3CA mutations are classified as tier IA by the ESMOs’ ESCAT scale. Recommendation for PIK3CA mutation profiling in primary tumor tissue, metastasis or plasma was confirmed in 2023.	10.1016/j.annonc.2021.09.019 and 10.1016/j.annonc.2020.09.010 and 10.1159/000531579
cfDNA PIK3CA mutation	NCT02379247, HER2-negative heavily pre-treated patients	Alpesilib might be applied in more HER2-negative patients because its application demonstrated that in combination with nab-paclitaxel a prolonged PFS could be achieved in heavily pre-treated patients with PIK3CA mutation in tumor or plasma compared to PIK3CA wildtype patients	10.1158/1078-0432.CCR-20-4879
**(2) CTCs**			
CTC PIK3CA mutations	MBCs	16% to 33% of all MBCs were reported to harbor PIK3CA mutant CTCs	10.1016/j.molonc.2014.12.001 and 10.1016/j.molonc.2013.07.007
**(5) multiple blood analytes**			
PIK3CA mutation in cfDNA and CTCs	MBCs	PIK3CA mutational status was found concordant in cfDNA and CTCs isolated from the same sample from MBC patients	10.1002/1878-0261.12540

**Table 10:** Liquid Biopsies for therapy guidance towards/against endocrine therapy.

Decision for/against Endocrine Therapy			
Specific Analyte	Clinical Setting	Conclusion	Reference
**(1) cfDNA**			
cfDNA ESR1 mutations	SoFEA trial, HR-positive MBC patients	Direct comparison of fulvestrant (SERD) with exemestane (AI), showed a significantly prolonged PFS using fulvestrant compared to exemestane in ESR1 mutant HR-positive MBC patients	10.1200/JCO.2016.67.3061
cfDNA ESR1 mutations	SoFEA and EFECT trial	OS benefit for ESR1 mutant MBC patients treated with fulvestrant compared to exemestane	10.1158/1078-0432.CCR-20-0224
cfDNA ESR1 mutations	PADA-1 trial	Longitudinal monitoring via ESR1 mutation detection in the plasma under AI treatment and switch to fulvestrant plus CDK4/6i compared to continuation of AI after emergence of ESR1 mutations without radiographic evidence for progression increased the PFS from 5.7 months to 11.9 months.	10.1016/S1470-2045(22)00555-1.
cfDNA ESR1 mutations	EMERALD trial, ER-positive/HER2-negative MBC in the second or more therapy line after progression under CDK4/6i and one previous chemotherapy line at maximum	Elacestrant was recently shown to significantly increase the PFS compared to standard endocrine monotherapy. This effect was shown for both, ESR1 mutant and ESR1 wild-type patients. The hazard ratio however, showed a greater effect of PFS prolongation from elacestrant compared to fulvestrant in ESR1 mutant patients compared to all patients, independent of their ESR1 status.	10.1200/JCO.22.00338
cfDNA ESR1 mutations	ER-positive/HER2-negative MBCs at the time of recurrence or progression on endocrine therapy	Testing for the emergence of ESR1 mutations is now recommended by the ASCO. Blood-based ESR1 mutation detection is preferred over tumor tissue testing due to the higher sensitivity. In HR-positive/HER2-negative MBC patients with prior CDK4/6i therapy and presence of ESR1 mutation in blood or tissue, elacestrant is recommended by the ASCO.	10.1016/S1470-2045(20)30444-7 and 10.1200/JCO.23.00638
cfDNA ESR1 promotor methylation		Methylation of the ESR1 promotor in cfDNA might become relevant for selection of an endocrine therapy	10.1158/1078-0432.CCR-17-1181
**(2) CTCs**			
CTC ESR1 promotor methylation		Methylation of the ESR1 promotor in CTCs might become relevant for selection of an endocrine therapy	10.1158/1078-0432.CCR-17-1181

**Table 11:** Liquid Biopsies for therapy guidance towards/against AKT inhibition.

Decision for/against AKT Inhibition			
Specific Analyte	Clinical Setting	Conclusion	Reference
**(1) cfDNA**			
cfDNA AKT1 mutation	plasmaMATCH trial, cohort C, ER-positive/HER2-negative MBC patients	Patients with AKT1 mutation in the cfDNA received capivasertib plus fulvestrant and this cohort met or exceeded the target number of responses with 4/18 patients	10.1016/S1470-2045(20)30444-7
cfDNA PIK3CA, AKT1 or PTEN alterations	PAKT trial, metastatic TNBC	Addition of capivasertib to paclitaxel compared to paclitaxel alone correlated with a prolonged PFS and OS, especially in patients with PIK3CA, AKT1 or PTEN alterations.	10.1200/JCO.19.00368

**Table 12:** Liquid Biopsies for therapy guidance towards/against Tyrosine receptor kinase (TRK) inhibition.

Decision for/against Tyrosine Receptor Kinase (TRK) Inhibition			
Specific Analyte	Clinical Setting	Conclusion	Reference
**(1) cfDNA**			
cfDNA NTRK1/2/3 fusion	BC patients	Trk inhibitors for BC patients with NTRK fusions are recommended. FDA approved blood-based evaluation of NTRK1/2/3 fusions in cfDNA available.	10.1159/000531579 and 10.1016/j.annonc.2020.09.010 and 10.1200/JCO.22.01063

**Table 13:** Liquid Biopsies for therapy guidance towards/against androgen receptor inhibition.

Decision for/against Androgen Receptor Inhibition			
Specific Analyte	Clinical Setting	Conclusion	Reference
**(2) CTCs**			
AR + CTCs	metastatic TNBC patients	AR protein expression analysis on CTCs in the blood might be usable as predictive marker for anti-AR therapy.	10.1002/ijc.32209
CTC AR_v7 mRNA	early TNBC patients	CTC mRNA analysis showed a minority of early TNBC patients to potentially benefit from anti-AR therapy based on AR_v7 transcript expression.	10.3389/fonc.2020.01658

**Table 14:** Liquid Biopsies for therapy guidance towards/against CDK4/6i.

Decision for/against CDK4/6i			
Specific Analyte	Clinical Setting	Conclusion	Reference
**(1) cfDNA**			
Tumor fraction in cfDNA	MBCs, before CDK4/6i initiation	Correlation of tumor fraction in cfDNA with PFS but not OS	10.1038/s41467-023-36801-9
cfDNA PIK3CA mutation	MBCs, before CDK4/6i initiation, *n* = 30 or in the MONALEESA-7 trial	potential of plasma PIK3CA mutations before CDK4/6i as predictive markers	10.1016/j.phrs.2020.105241 and 10.1200/PO.20.00445
cfDNA KRAS mutation	MONALEESA-7 trial, MBCs, before CDK4/6i initiation	Patients treated with palbociclib and fulvestrant with baseline KRAS mutations had a worse median PFS compared to patients with KRAS wild-type	10.1200/PO.20.00445
cfDNA RB1 mutation	PALOMA-3 trial, MBCs, before CDK4/6i initiation	patients with RB loss (17.3% prevalence) at baseline had a significantly worse PFS under palbociclib plus fulvestant compared to RB wild-type patients	10.1093/jnci/djaa087
cfDNA RB-LOH signature	245 patients treated with ET + CDK4/6i from two independent cohorts	RB-LOH signature, consisting of 224 copy number features in the entire cfDNA genome showed a strong correlation with poor response and poor survival following CDK4/6i plus endocrine therapy	10.1038/s41467-023-36801-9
**(2) CTCs**			
Single CTC RB1 transcript expression	within the TREnd trial, small cohort of MBC before Palbociclib	Gene expression regarding RB1 in single CTCs revealed a prolonged PFS	10.1186/s13058-021-01415-w
**(3) EVs**			
EV CDK4 mRNA expression	40 HR-positive/HER2-negative advanced BC patients receiving palbociclib plus endocrine therapy, at baseline	High mRNA expression levels of CDK4 in EVs correlated significantly with a longer PFS	10.1007/s10549-019-05365-y

**Table 15:** Genomic alterations of strong clinical significance (Tier I Level A by AMP/ASCO/CAP Variant Categorization [210]) predictive for response to FDA-approved drugs in breast cancer (according to OncoKB Level of Evidence 1, 2 and R1 [209]). ESCAT scale according to [208,256]. ASCO recommendation according to [271,331] and to [292]. AGO guidelines according to [98,148].

Gene	Alteration	Cancer Type	Drugs	ESCAT Scale 2019/2020	ASCO 2022	AGO 2023
PIK3CA	C420R, E542K, E545A, E545D, E545G, E545K, Q546E, Q546R H1047L, H1047R, H1047Y and other oncogenic mutations	Breast Cancer	Alpelisib + Fulvestrant	IA	recommended	++
ERBB2	Amplification	Breast Cancer	Trastuzumab, Trastuzumab + Chemotherapy	IA		
ERBB2	Amplification	Breast Cancer	Trastuzumab Deruxtecan	IA		
ERBB2	Amplification	Breast Cancer	Trastuzumab + Pertuzumab + Chemotherapy	IA		
ERBB2	Amplification	Breast Cancer	Trastuzumab + Tucatinib + Capecitabine	IA		
NTRK1	Fusion	All Solid Tumors	Larotrectinib	IC	recommended	+
NTRK2	Fusion	All Solid Tumors	Larotrectinib	IC	recommended	+
NTRK3	Fusion	All Solid Tumors	Larotrectinib	IC	recommended	+
NTRK1	Fusion	All Solid Tumors	Entrectinib	IC	recommended	+
NTRK2	Fusion	All Solid Tumors	Entrectinib	IC	recommended	+
NTRK3	Fusion	All Solid Tumors	Entrectinib	IC	recommended	+
	Microsatellite Instability-High	All Solid Tumors	Pembrolizumab	IC	recommended	+
	Tumor Mutational Burden-High	All Solid Tumors	Pembrolizumab	IC	recommended	
ESR1	D538, E380, L469V, L536, S436P, Y537, V422del	Breast Cancer	Elacestrant	IIA	not recommended in 2022, but recommended in 2023	+
ERBB2	Amplification	Breast Cancer	Ado-Trastuzumab Emtansine			
ERBB2	Amplification	Breast Cancer	Lapatinib + Capecitabine, Lapatinib + Letrozole			
ERBB2	Amplification	Breast Cancer	Margetuximab + Chemotherapy			
ERBB2	Amplification	Breast Cancer	Neratinib, Neratinib + Capecitabine			
BRAF	V600E	All Solid Tumors (excluding Colorectal Cancer)	Dabrafenib + Trametinib			
NTRK1	G595R	All Solid Tumors	Resistance to Larotrectinib			
NTRK3	F617L	All Solid Tumors	Resistance to Larotrectinib			
NTRK3	G623R	All Solid Tumors	Resistance to Larotrectinib			
NTRK3	G696A	All Solid Tumors	Resistance to Larotrectinib			
RET	Fusion	All Solid Tumors (excluding Thyroid Cancer, Non-Small Cell Lung Cancer)	Selpercatinib			

**Table 16:** Liquid Biopsies for therapy monitoring.

Therapy Monitoring			
Specific Analyte	Clinical Setting	Conclusion	Reference
**(1) cfDNA**			
cfDNA methylation (9 marker)	TBCRC 005 study, MBCs, under therapy	9-marker cfDNA methylation assay was shown to forecast disease progression three months earlier than radiographic staging in MBC patients	10.1158/1078-0432.CCR-22-2128
genomic instability of cfDNA	25 MBC patients, at baseline, one week under therapy, three months after therapy initiation	More than 50% reduction in genomic instability number (GIN) from low-pass WGS of cfDNA at baseline to one week under therapy was shown to associate with the stable disease proven by staging after 3 months and also with OS. A rise in GIN from baseline to two weeks under therapy associated with poor response, evaluated three months after therapy initiation by staging.	10.3390/cancers13061331
cfDNA CNVs	HR-positive/HER2-negative MBC patients treated with CDK4/6i, at baseline and under therapy	comparison of z-scores at baseline and under therapy (z-score trajectories) has monitoring value	10.1002/1878-0261.12870
mean allele frequency dynamics in cfDNA	LOTUS and INSPIRE trials, MBCs treated with different therapy regimens, baseline to a time point under therapy	Mean allele frequency dynamics from baseline to a time point under therapy related to therapy response at the time of blood draw or to PFS and OS	10.1200/PO.20.00345 and 10.1200/PO.20.00345 and 10.1002/mgg3.1079 and 10.1038/s41523-021-00218-8 and 10.1016/S1470-2045(17)30450-3 and 10.1186/s40425-019-0541-0
ctDNA mutations	POSEIDON and SUMMIT trials, under therapy	In the POSEIDON and SUMMIT trials, early evaluation of ctDNA changes forecasted the radiologic treatment response and the emergence of specific mutations correlated with clinical drug resistance. Allele frequency of HER2 mutations in cfDNA decreased under pan-HER inhibitor neratinib, but increased upon radiographically proven progression.	10.1158/1078-0432.CCR-19-0508 and 10.1158/1078-0432.CCR-17-0900.
ctDNA in CSF and plasma	HER2-positive MBCs with brain metastases	dynamic changes in ctDNA in CSF and plasma under therapy revealed decreased allele frequencies in the plasma to be consistent with extra-CNS disease control and increased allele frequencies in the CSF to be related to poor treatment benefit in CNS	10.1136/esmoopen-2017-000253
ctDNA level	INSPIRE trial, TNBC patients and patients with other tumor entities, from baseline to six weeks under treatment with pembrolizumab	ctDNA level changes from baseline to six weeks under treatment forecasted the therapy benefit. In all patients who responded to therapy, ctDNA clearance was detected before visible radiological response.	10.1038/s43018-020-0096-5
cfDNA PIK3CA mutations	PALOMA-3 trial, palbociclib treated patients from baseline to two weeks	cfDNA PIK3CA mutation dynamics had significant monitoring value. Decrease in PIK3CA mutations in the cfDNA correlated significantly with increased PFS and long-term clinical benefit.	10.1038/s41467-018-03215-x
cfDNA level and mutations	ALCINA trial, at day 15/30 under palbociclib plus fulvestrant	cfDNA evaluation showed a decrease in all patients independent of their PFS. On day 30, undetectable cfDNA mutations (PIK3CA, TP53 and AKT1 studied) associated with improved PFS.	10.1186/s13058-021-01411-0 and 10.1038/s41388-020-1174-y
cfDNA ESR1 mutations	MBCs under first-line AI treatment	ESR1 mutation detection in the plasma revealed a direct association with progressive disease with a 100% specificity. ESR1 mutations were detectable prior to progression with median lead time of 110 days.	10.1186/s13058-020-01290-x
cfDNA ESR1 mutations	PADA-1 trial, under palbociclib and AI	Rising allele frequencies of cfDNA ESR1 mutations were used to identify patients with no radiographically proven progressive disease suitable for therapy switch of endocrine therapy. Significant clinical benefit with regard to PFS in case the therapy switch was conducted in patients with rising ESR1 mutations detectable under therapy	10.1016/S1470-2045(22)00555-1
**(2) CTCs**			
CTC count by CellSearch	3–5 or 6–8 weeks after initiation of therapy	It was shown that the CTC count itself by CellSearch evaluated 3–5 or 6–8 weeks after initiation of therapy was significantly associated with PFS and OS	10.1016/S1470-2045(14)70069-5.
CTC count	from baseline to a time point under therapy	A decrease in CTC counts from baseline to a time point under therapy was related to an increased PFS and OS. Persistently high CTC counts from baseline to under therapy, despite radiologically proven therapy response, associated with worse outcome	10.1158/1078-0432.CCR-05-2821 and 10.1158/1078-0432.CCR-05-1769
apoptotic CTCs	baseline to under therapy	number of apoptotic CTCs from baseline to under therapy revealed a 50% apoptotic CTC reduction to differentiate between patients showing stable versus progressive disease and in case the apoptotic CTC number decreased from baseline to under therapy by less than 10%, progressive disease was identified with 74% specificity	10.3390/cancers12041055
HER2+ CTCs	MBC patients treated with anti-HER2 treatment lapatinib	significant decrease in HER2-positive CTCs was only detected in MBC patients responding to anti-HER2 treatment with lapatinib, but not in patients progressing under lapatinib	10.1371/journal.pone.0123683
RANK-positive CTCs	MBCs treated with Denosumab, baseline to day 2	increase in RANK-positive CTCs from baseline to day 2 and persistence of RANK-positive CTCs was related to a longer time to progress of the bone metastasis	10.1038/s41598-020-58339-2
CTCs overexpressing EpCAM, MUC1 or HER2	under therapy in MBCs	The persistence of CTCs overexpressing EpCAM, MUC1 or HER2 transcripts under therapy in MBC patients correlated with shorter OS	10.1007/s10549-008-0143-x
CTCs overexpressing either EMT markers or the stem cell marker ALDH1	MBCs, at the staging time point	74% of all patients with progressive disease have CTCs overexpressing either EMT markers or the stem cell marker ALDH1 in contrast to only 10% of patients with stable disease	10.1186/bcr2333
CTC mRNA profile	MBCs, at the staging time point	overexpression of ERBB2, ERBB3, ERCC1 alone or in combination with AURKA in CTCs of MBCs was significantly more prevalent in patients showing progressive disease at the time of blood draw compared to patients with stable disease. Identification of CTCs with overexpression of ERBB2, ERBB3, ERCC1 alone or in combination with AURKA during therapy in MBCs was furthermore related to a shorter OS. ERBB2 overexpression in CTCs was related to therapy failure at the time of blood draw and to a reduced OS	10.18632/oncotarget.9528
CTC mRNA profile	MBCs, at the staging time point	Patients with progressive disease at the time of blood draw were more likely to have CTC overexpression signals than patients with stable disease. Two different gene expression patterns in CTCs were shown for patients with progressive disease, but a homogeneous expression pattern in patients with stable disease	10.1373/clinchem.2016.269605
ERBB2 and/or ERBB3 overexpression in CTCs	MBCs, at the staging time point	ERBB2 and/or ERBB3 overexpression in CTCs was significantly correlated with progressive disease at the time of blood draw	10.1373/clinchem.2017.283531
**(4) other blood analytes**			
CEA, CA 15-3, and CA 27-29	BC	The circulating proteins CEA, CA 15-3, and CA 27-29 were recommended for therapy monitoring in 2015 by the ASCO	10.1200/JCO.2015.61.1459
**(5) multiple blood analytes**			
CTC and EV mRNA profiles	MBCs, at the staging time point	Stronger correlation of ERBB2 and ERBB3 signals in CTCs and EVs with disease progression was identified compared to ERBB2 and ERBB3 signals in CTCs alone, revealing a synergistic value of CTCs and EVs for therapy monitoring. mTOR overexpression signals in EVs of MBCs under therapy was related to consecutive therapy failure while mTOR overexpression in CTCs was related to patients showing therapy response over at least six months	10.1373/clinchem.2017.283531
ctDNA, CTC, CA 15-3	MBCs	ctDNA evaluation was shown to have a higher sensitivity and higher correlation with tumor burden compared to CA 15-3 and CTC evaluations.	10.1056/NEJMc1306040
cfDNA, CTC genomic DNA, CTC mRNA and EV mRNA	MBCs, at the staging time point	Additive value of these analytes in treatment monitoring. Presence of either ERBB3 overexpression signals or ERBB2 overexpression signals in CTCs were related significantly to the staging result. Combined evaluation of ERBB3 in all three analytes associated with therapy response. Dynamics from one time point to the next time point were more informative than single time point evaluations. Overexpression signals in EVs were the most dynamic ones during therapy and newly occurring ERCC1 overexpression signals in EVs from one time point to the next had a specificity of 97% but sensitivity of 18% to determine therapy response. The accuracy for detecting disease progression was 70% and 66% for PIK3CA and ESR1 variant appearances and the combined evaluation of ESR1 or PIK3CA allele frequency development was significantly correlated with disease progression.	10.3390/cells10020212

## Abbreviations

AI: aromatase inhibitor; AR: androgen receptor; ASCO: American Society of Clinical Oncology; BC: breast cancer; CDK4/6i: Cyclin dependent kinase 4/6 inhibition; CDx: companion diagnostic; CEN: Committee for Standardization; cfDNA: cell-free DNA; cfRNA: cell-free RNA; CHIP: clonal hematopoiesis of undetermined potential; CK: cytokeratin; CNV: copy number variation; CTC: circulating tumor cell; ctDNA: circulating tumor DNA; ctRNA: circulating tumor RNA; CTX: chemotherapy; d(d)PCR: digital (droplet) PCR; DCIS: ductal carcinoma in situ; DDSF: distant disease-free survival; DFS: disease-free survival; DTC: disseminated tumor cell; EMT: epithelial-mesenchymal transition; ESCAT: ESMO Scale for Clinical Actionability of molecular Targets; ESMO: European Society for Medical Oncology; ET: endocrine therapy; EV: extracellular vesicle; g: germline; gDNA: genomic DNA; GIN: genomic instability number; HER2: human epidermal growth factor receptor; HR: hormone receptor; ICI: immune checkpoint inhibitor; LB: liquid biopsy; LOE: level of evidence; MBC: metastatic breast cancer; miR/miRNA: micro RNA; MMR-D: mismatch repair deficiency; MRD: minimal residual disease; mRNA: messenger RNA; MSI: microsatellite instability; NACT: neoadjuvant chemotherapy; NGS: next-generation sequencing; NLR: neutrophil-to-lymphocyte ratio; OS: overall survival; PBMC: peripheral blood mononuclear cells; pCR: pathological complete remission; PCR: polymerase chain reaction; PD1: programmed cell death protein 1; PDL1: programmed cell death ligand 1; PFS: progression-free survival; PPV: positive predictive value; qPCR: quantitative PCR; SERD: selective ER degrader; SERM: selective ER modulator; SNV: single nucleotide variation; sT1K: serum thymidine kinase 1; TCR: T-cell receptor; TILs: tumor-infiltrating lymphocytes; TMB: tumor mutational burdern; TNBC: triple negative breast cancer; TX: therapy; WES: whole exome sequencing; WGS: whole genome sequencing
